# Cyclin-Dependent Kinases (CDKs) and the Human Cytomegalovirus-Encoded CDK Ortholog pUL97 Represent Highly Attractive Targets for Synergistic Drug Combinations

**DOI:** 10.3390/ijms23052493

**Published:** 2022-02-24

**Authors:** Markus Wild, Friedrich Hahn, Nadine Brückner, Martin Schütz, Christina Wangen, Sabrina Wagner, Mona Sommerer, Stefan Strobl, Manfred Marschall

**Affiliations:** 1 Institute for Clinical and Molecular Virology, Friedrich-Alexander Universität Erlangen-Nürnberg (FAU), Schlossgarten 4, 91054 Erlangen, Germany; markus.wild@uk-erlangen.de (M.W.); friedrich.hahn@uk-erlangen.de (F.H.); nadine.brueckner@fau.de (N.B.); martin.schuetz@uk-erlangen.de (M.S.); christina.wangen@uk-erlangen.de (C.W.); sabrina.wagner@uk-erlangen.de (S.W.); monas.sommerer@fau.de (M.S.); 2 4SC AG/4SC Discovery GmbH, Fraunhoferstraße 22, 82152 Planegg-Martinsried, Germany; stefan.strobl@biontech.de; 3 BioNTech SE, Am Klopferspitz 19a, 82152 Planegg-Martinsried, Germany

**Keywords:** human cytomegalovirus, antiviral drug, drug combinations, antiviral drug synergism, host-directed antiviral (HDA), direct-acting antiviral (DAA), pharmaceutical kinase inhibitor (PKI), cyclin-dependent kinase (CDK), viral CDK pUL97 (vCDK), new synergistic drug-target combinations

## Abstract

Human cytomegalovirus (HCMV) is a pathogenic human herpesvirus associated with serious, potentially life-threatening symptoms in the immunocompromised or immunonaïve host. The limitations encountered by antiviral therapy options currently available include a narrow panel of accessible targets, the induction of viral drug resistance as well as severe drug dosage-mediated side-effects. Improved drug-targeting strategies to resolve these issues are the focus of our investigations. In particular, pharmaceutical kinase inhibitors (PKIs), either directed to host kinases or directed to the viral protein kinase pUL97, have been considered to overcome these restrictions. Recently, we reported the identification of a synergistic combination of two PKIs directed to host cyclin-dependent kinase 7 (CDK7) and viral CDK ortholog pUL97. Here, we substantiate these findings with the following results: (i) true drug synergy was exhibited by various chemical classes of PKI pairs directed to pUL97 and CDK7; (ii) no putative amplification of cytotoxicity by these drug combinations was observed; (iii) a reduction in drug dosage levels for synergistic combinations was defined on a quantitative basis and compared to monotreatments; (iv) the quantities of target proteins CDK7 and pUL97 expressed in HCMV-infected cells were assessed by confocal imaging, indicating a strong down-modulation of CDK7 levels as a result of synergistic drug treatment; (v) the functional importance of these target kinases, both binding to cyclin H, was illustrated by assessing HCMV replication under the viral genomic deletion of ORF-UL97 or cellular cyclin knock-out; (vi) new combinations of HCMV-specific drug synergy were demonstrated for solely host-directed treatments using PKIs against CDK2, CDK7, CDK8 and/or CDK9 and (vii) a triple PKI combination provided further support for the synergy approach. With these combined findings, this study highlights the potential of therapeutic drug combinations of approved, developmental and preclinical PKIs for expanding future options for anti-HCMV therapy.

## 1. Introduction

Currently available antiviral drugs are mainly based on direct-acting antivirals (DAAs), with only a few exceptions, such as interferon-based treatments and some host-directed antiviral (HDA) approaches in AIDS therapy. These DAAs, however, share certain disadvantages that often substantially limit their therapeutic benefit: (i) a common tendency to induce drug-resistant virus mutants; (ii) inhibitory activity in a virus-specific, but not in a broader manner spanning a spectrum of viruses and (iii) a limited clinical efficacy in several cases. Generally, antiviral DAA-type drugs are continuously subject to a developmental process of refinement and a stepwise replacement by next-generation candidates. A major improvement may be achieved by expanding options of antiviral treatment by integrating HDAs and drug combination treatment schemes. Very recently, the use of pharmaceutical kinase inhibitors (PKIs) in antiviral drug research, originally developed for cancer and inflammatory disease therapy, showed very promising success [1,2,3,4,5]. As a current example, the PKI maribavir (MBV) has been approved for the therapy of human cytomegalovirus (HCMV) post-transplant disease (FDA, Nov. 2021) after the successful termination of clinical phase III studies ([6,7]; NCT02931539, NCT02927067, NCT00497796, NCT00411645). Thus, MBV constitutes the first PKI in antiviral treatment. Concerning the further application of PKIs, the area of antiherpesviral prophylaxis and therapy appears especially promising. This is due to an increasing amount of published data indicating that for some human pathogenic herpesviruses, potential target kinases of antiviral PKIs are not restricted to virus-encoded protein kinases, but also include virus-supportive regulatory host kinases, thus representing potential new antiviral targets.

Human cytomegalovirus (HCMV) represents the prototype species of *Betaherpesvirinae* and an opportunistic human pathogen with a predominant, worldwide distribution. Seroprevalence ranges between 40% and 95% in the adult human population, dependent on socio-geographic factors [8,9]. In the immunocompetent host, infections with HCMV typically cause only mild symptoms or remain asymptomatic [10]. In immunocompromised or immunonaïve individuals, however, HCMV infection can lead to significant morbidity and mortality, specifically in patients under antitumoral chemotherapy, stem cell/organ transplantation or coinfection with human immunodeficiency virus type 1 (HIV-1). Moreover, congenital HCMV infection (cCMV), which has been underestimated for a long time, is the main infection-based risk during pregnancy [11,12,13]. Specifically, cCMV is responsible for a variety of symptoms from mild to severe or even life-threatening in the unborn or infants, mainly manifesting as acute or late-onset embryonal developmental defects [14]. Notably, among the current repertoire of approved anti-HCMV drugs, only a small selection can be used for cCMV therapy, prevention and control. For the most part, the approved drugs are nucleoside/nucleotide or pyrophosphate analogs which intervene with the activity of viral genome replication, i.e., ganciclovir (GCV), its oral prodrug valganciclovir (VGCV), foscarnet (FOS) and cidofovir (CDV) [15,16]. Recently, letermovir (LMV, Prevymis^®^), an inhibitor of the viral terminase, has been clinically approved, albeit exclusively for the prophylaxis of HCMV infection in recipients of hematopoietic stem cell transplantation [17,18]. Now, after decades of development, maribavir (MBV/Livtencity^®^) could also be added to the panel of available anti-HCMV drugs. Nevertheless, this panel still faces substantial limitations, such as the induction of viral drug resistance and, in the case of VGCV standard therapy, severe side effects including nephrotoxicity, myelotoxicity and anemia, often limiting their therapeutic compatibility and use in long-term treatments [19,20].

Our approach focuses on novel drug application schemes and targeting strategies, i.e., the development of mechanistically new antiviral drug candidates and so far unexploited targeting approaches. The HCMV-specific antiviral potential of the CDK7 inhibitor LDC4297 has already been demonstrated in our previous studies. Specifically, its mode of action (MoA) has been characterized as a complex inhibitory effect resulting from interference with CDK7 activity, i.e., an inhibition of the immediate early phase of viral replication and cell cycle modulation through altered Rb phosphorylation [21]. For the HDA LDC4297, a strong synergistic drug interaction with DAA MBV was described in our previous work [1]. This synergism between two PKIs was further characterized in the current study, including investigations into its molecular mechanistic parameters. Furthermore, we extended our investigations into the promising antiviral activity of additional CDK inhibitors, vCDK/pUL97 inhibitors and, in particular, new synergistic drug combinations.

## 2. Results

### 2.1. Evaluation of Antiviral Efficacy and Drug-Induced Cytotoxicity of Selected CDK Inhibitors and Drug Combinations

For a comprehensive analysis of the antiviral activity of CDK inhibitors and vCDK/pUL97 inhibitors, we selected a broad panel of PKIs, the majority of which are currently investigated at preclinical and clinical levels (https://www.ppu.mrc.ac.uk/list-clinically-approved-kinase-inhibitors; last accessed on 26 January 2022; [22,23]). We focused on PKIs targeting human CDKs 2, 7, 8, 9 as well as vCDK/pUL97, since these kinases have all been shown to exert a relevant regulatory role for efficient HCMV replication [1,5,21,24,25,26,27,28,29,30,31]. As an important result, a number of PKIs, i.e., maribavir, Gö6976, Ax7396, Vi7392, CDK2 Inh II, LDC4297, SEL120 and THAL-SNS032, showed pronounced anti-HCMV activity in a range of EC_50_ values between 0.009 µM and 6.33 µM (Table 1, upper panel). Drug-induced cytotoxicity was assessed by neutral red assay (NRA) performed with mock-infected primary human foreskin fibroblasts (HFFs) and was expressed as the half maximal cytotoxicity (CC_50_). For all of the mono-selective PKIs mentioned above, the levels of cytotoxicity were found to be clearly separate from concentrations relevant for antiviral activity (Table 1, upper panel; Figure S1). This was not the case, however, for a number of other clinically relevant PKIs, i.e., CVT-313, SY1365, samuraciclib, AZD4573, dinaciclib, riviciclib and CYC065, which induced higher levels of cytotoxicity on HFFs and were thus not further investigated in this study (Table 1, lower panel; Figure S2).

In addition to this assessment of drug-induced cytotoxicity by single compounds, specific focus was given to a putative amplification of cytotoxicity in drug combination settings. Such a combination-specific enhancement of cytotoxicity could theoretically overlay antiviral synergistic effects, as later measured by the Bliss checkerboard and Loewe fixed-dose assay systems. To this end, all drug combinations analyzed for antiviral drug–drug interaction were additionally screened for cytotoxic effects using the NRA (Table 2; Figure S2). Signs of combination-specific enhancement of cytotoxicity only became apparent in rare cases. The antiviral activity of combined drug treatments was exhibited in an EC_50_ range between 0.01 µM and 3.3 µM (Table 2; see also Table 3, Table 4, Table 5, Table 6 and Table 7 for further details). It has to be emphasized that an assessment of drug–drug interaction should generally not be based on single-dose escalations, i.e., relying solely on the EC_50_ values, since gradual dose-effect curves are essential for a reliable determination using the Bliss and Loewe systems [32] (see Section 2.2. and Section 2.3). Concerning cytotoxicity, it was only in two cases, for SEL120 + THAL-SNS032 and SEL120 + LDC4297, that the EC_50_ and CC_50_ values showed a narrow window of selectivity index (SI), of 2 and 4, respectively. In other cases, such as Gö6976 + LDC4297, Ax7396 + LDC4297 or MBV + THAL-SNS032, only high concentrations of drug combinations resulted in cytotoxicity, as indicated by SI values of 90, 47 and 37, respectively. This finding allowed for a safe evaluation of antiviral activity for all selected drug combinations (Figures S1 and S2, Table 2). An example of a triple drug combination, i.e., MBV + LDC4297 + SEL120 (see Section 2.7 for antiviral assessment), was also examined for cytotoxicity and showed no enhancement (SI of 270; Table 2, lower panel). Thus, for the majority of the analyzed PKIs, the levels of drug-induced cytotoxicity could be clearly separated from concentrations of true antiviral activity.

### 2.2. Assessment of Drug–Drug Interactions of Purely Host-Directed PKI Combinations

**Figure 1:**
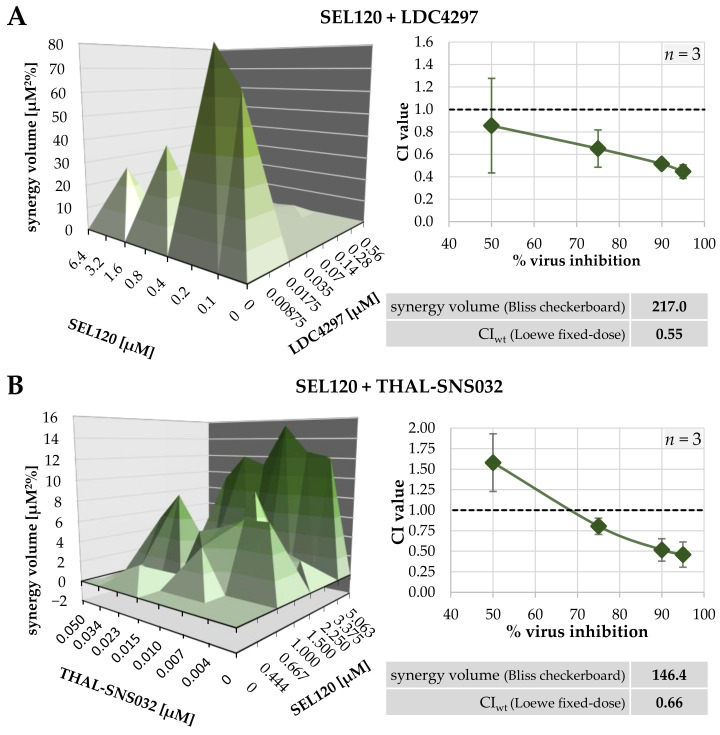
Depiction of two synergistic interactions between host-directed PKIs, evaluated via Bliss independence checkerboard assay (left) and Loewe additivity fixed-dose assay (right): (**A**) SEL120 + LDC4297, (**B**) SEL120 + THAL-SNS032. The inserted tables shaded in grey give the sum of positive and negative 95% confidence interval synergy volumes (SV) [µM^2^%], as well as weighted combination index values (CI_wt_) for these two drug combinations (see Table 3 and Table 4 for detailed results).

Recently, we reported the strong synergistic anticytomegaloviral potential of the combination of an inhibitor of the viral CDK ortholog pUL97 (MBV) and a CDK7-specific inhibitor (LDC4297, pyrazolotriazine class) [1]. Here, we investigated drug–drug interactions of further combinations, in this case purely based on pairs of host-directed antivirals (HDAs). To this end, inhibitors targeting the host kinases CDK2 (CDK2 Inh II, CVT-313), CDK7 (LDC4297), CDK8 (SEL120) and CDK9 (THAL-SNS032) were assessed for anti-HCMV activity in the context of either single or combination treatments (Figure 1). In the single treatment settings, all PKIs showed a pronounced level of antiviral activity within the low micromolar to submicromolar range (Table 1). Importantly, we identified two new cases of true anti-HCMV drug synergy for HDA-exclusive combinations, i.e., CDK7 (LDC4297) + CDK8 (SEL120) and CDK8 (SEL120) + CDK9 (THAL-SNS032) (Figure 1, Table 3 and Table 4; for THAL-SNS032 see additional data in Hahn et al., 2021 [33]). The classification of drug–drug interaction was based on assessment with two independent analysis systems, i.e., the Bliss checkerboard assay (Figure 1, left panels; Table 3) and Loewe additivity fixed-dose assay (Figure 1, right panels; Table 4), measured as synergy volume (SV, µM^2^%) and weighted combination index (CI_wt_), respectively. The SV of these synergistic combinations ranged from 148.4 to 217 µM^2^% (strongly synergistic) and the CI_wt_ values from 0.55 ± 0.06 to 0.66 ± 0.1. Thus, the CDKs 7, 8 and 9 could be nominated as suitable targets for synergistic anti-HCMV drug combinations with a purely host-directed MoA. Interestingly, however, another drug combination exhibited an antagonistic effect, i.e., CDK2 (CDK2 Inh II) + CDK7 (LDC4297) (Table 3). The mechanistic basis of this negative interference between inhibitors of CDK2 and CDK7 in anti-HCMV activity is not yet known. 

**Table 3:** Combinatorial anti-HCMV drug assessment using the Bliss independence checkerboard assay.

Drug Combination	95% Confidence Interval Synergy Volume [µM^2^%] ^b^			Drug Interaction Type
	Replicates ^a^	Positive	Negative	
SEL120 + LDC4297	1	**217.0**	**0**	strongly synergistic
SEL120 + THAL-SNS032	1	**148.4**	**−2.0**	strongly synergistic
CDK2 Inh II + LDC4297	1	**0**	**−251.3**	antagonistic

^a^ Individual experimental replicates were performed in biological triplicates. ^b^ The synergy volume [µM^2^%] values defining the drug interaction type are in bold print. The types of drug interaction were defined as follows: values below −100, antagonistic; −100 to +50, additive; +50 to +100, moderately synergistic; above +100, strongly synergistic [1,32].

**Table 4:** Combinatorial anti-HCMV drug assessment using the Loewe additivity fixed-dose assay.

Drug Combination	Ratio	Replicates ^a^	CI Values Extrapolated at x Virus Inhibition				CI_wt_ ^b^
			50%	75%	90%	95%	
SEL120 + LDC4297	10:1	3	0.86 ± 0.42	0.65 ± 0.17	0.51 ± 0.03	0.45 ± 0.06	**0.55** ± 0.06
SEL120 + THAL-SNS032	50:1	3	1.58 ± 0.35	0.80 ± 0.10	0.52 ± 0.14	0.46 ± 0.15	**0.66** ± 0.10

^a^ Individual experimental replicates were performed in biological duplicates. ^b^ The CI_wt_ values defining the drug interaction type are in bold print and were calculated as 0.1 × CI_50_ + 0.2 × CI_75_ + 0.3 × CI_90_ + 0.4 × CI_95_ and defined as follows: 0 to 0.3, strongly synergistic; 0.3 to 0.7, synergistic; 0.7 to 0.85, moderately synergistic; 0.85 to 0.9, slightly synergistic; 0.9 to 1.1, (nearly) additive; 1.1 to 1.2, slightly antagonistic; 1.2 to 1.45, moderately antagonistic; 1.45 to 3.3, antagonistic; 3.3 to > 10, strongly antagonistic [1,32].

### 2.3. Assessment of Drug–Drug Interactions of PKI Combinations Directed to Both Host CDKs and vCDK/pUL97

**Figure 2:**
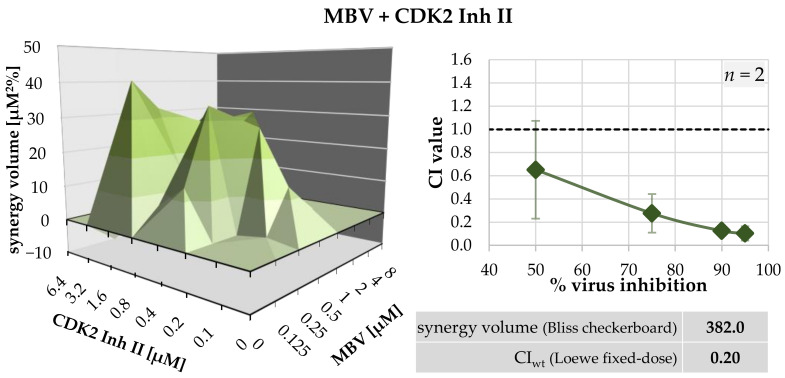
Depiction of a synergistic interaction between MBV + CDK2 Inh II, representing a pair of virus- and host-directed PKIs. Evaluation was performed using the Bliss independence checkerboard assay (left) and Loewe additivity fixed-dose assay (right). The inserted table shaded in grey gives the sum of positive and negative 95% confidence interval SV [µM^2^%] as well as CI_wt_ (see Table 5 and Table 6 for detailed results).

A profound characterization of the antiviral properties of MBV and LDC4297 in vitro and in vivo has been reported in our previous studies [5,21,24,34,35,36,37,38,39,40,41]. An important hallmark was the finding that MBV + LDC4297 combinatorial treatment of HCMV replication in cell culture models exhibited a synergistic effect that was clearly distinct from additive or antagonistic effects produced by other compound combinations [1]. Given the plausible option that the synergism of MBV + LDC4297 might be restricted to the characteristics of these two chemical compounds, but does not extend to other inhibitors of the two targets CDK7 and pUL97, we addressed this point in more detail. We analyzed four different inhibitors of vCDK/pUL97 derived from distinct chemical classes, i.e., MBV (benzomidazole riboside), Ax7396 (quinazoline), Gö6976 (indolocarbazole) and Vi7392 (quinazoline; for chemical structures, see Steingruber and Marschall, 2020 [5]). These vCDK/pUL97 inhibitors were analyzed in parallel for synergy with the CDK7 inhibitor LDC4297. Importantly, true drug synergy was verified for all cases of vCDK/pUL97 + CDK7 inhibitor combinations, with CI_wt_ values consistently in a narrow range between 0.36 ± 0.22 and 0.62 ± 0.12 (Table 5). This finding clearly confirmed a constant synergistic effect via the combined targeting of these two protein kinases, independent of the drugs’ chemical classes. Moreover, we identified a new promising targeting option, namely vCDK/pUL97 (MBV) + CDK2 (CDK2 Inh II). Both evaluation systems, the Loewe additivity fixed-dose assay (Table 5) and Bliss independence checkerboard (Table 6, Figure 2), indicated synergism for this drug combination, with a positive SV value of 394.3 µM^2^% (strongly synergistic) and a CI_wt_ value of 0.20 ± 0.13. Thus, this specific drug combination based on vCDK/pUL97 + CDK2 targeting also suggests new options for antiviral pharmacological development.

**Table 5:** Combinatorial anti-HCMV drug assessment using the Loewe additivity fixed-dose assay.

Drug Combination	Ratio	Replicates ^a^	CI Values Extrapolated at x Virus Inhibition				CI_wt_ ^b^
			50%	75%	90%	95%	
MBV + LDC4297 ^c^	100:1	4	0.37 ± 0.41	0.29 ± 0.23	0.30 ± 0.18	0.44 ± 0.36	**0.36** ± 0.22
Ax7396 + LDC4297	100:1	3	0.94 ± 0.47	0.68 ± 0.07	0.57 ± 0.18	0.55 ± 0.26	**0.62** ± 0.12
Gö6976 + LDC4297	100:1	3	0.70 ± 0.66	0.34 ± 0.14	0.37 ± 0.18	0.42 ± 0.23	**0.42** ± 0.14
Vi7392 + LDC4297	100:1	3	0.49 ± 0.12	0.51 ± 0.11	0.55 ± 0.14	0.59 ± 0.17	**0.55** ± 0.13
MBV + CDK2 Inh II	1:1	2	0.65 ± 0.56	0.28 ± 0.24	0.13 ± 0.09	0.10 ± 0.01	**0.20** ± 0.13

^a^ Individual experimental replicates were performed in biological duplicates. ^b^ The CI_wt_ values defining the drug interaction type are in bold print and were calculated as 0.1 × CI_50_ + 0.2 × CI_75_ + 0.3 × CI_90_ + 0.4 × CI_95_ and defined as follows: 0 to 0.3, strongly synergistic; 0.3 to 0.7, synergistic; 0.7 to 0.85, moderately synergistic; 0.85 to 0.9, slightly synergistic; 0.9 to 1.1, (nearly) additive; 1.1 to 1.2, slightly antagonistic; 1.2 to 1.45, moderately antagonistic; 1.45 to 3.3, antagonistic; 3.3 to >10, strongly antagonistic [1,32]. ^c^ For additional data, see also [1].

**Table 6:** Combinatorial anti-HCMV drug assessment using the Bliss independence checkerboard assay.

Drug Combination	Cell type/Virus	Replicates ^a^	95% Confidence Interval Synergy Volume [µM^2^%] ^b^		Drug Interaction Type
			Positive	Negative	
MBV + CDK2 Inh II	HFF/HCMV	1	**394.3**	**−12.3**	strongly synergistic

^a^ Individual experimental replicates were performed in biological triplicates. ^b^ The synergy volume [µM^2^%] values defining the drug interaction type are in bold print. The types of drug interaction were defined as follows: values below −100, antagonistic; −100 to +50, additive; +50 to +100, moderately synergistic; above +100, strongly synergistic [1,32].

When combined, the present data further underline that synergistic antiviral drug combinations, such as MBV and LDC4297, may be successfully utilized in cotreatment regimens. A benefit of such synergistic combination treatments might be a substantial reduction in individual drug dosages while still achieving an optimal treatment efficacy. As an illustration, in vitro drug dosage reductions of identified synergistic combinations were determined (Table 7). The underlying calculation is based on data shown in Table 5, so that combinations of CDK7 inhibitor LDC4297 with each of the four pUL97 inhibitors (MBV, Ax7396, Gö6976 and Vi7392) are displayed. Table 7 shows EC_50_ and EC_90_ values for the drug combinations (grey shading) and single drugs (white). Note that the EC_50_ or EC_90_ values of the combinations were based on the cooperative activity of the two individual compounds in the respective combination setting (e.g., 0.043 µM MBV + 0.0004 µM LDC4297 resulted in 50% inhibition of viral replication). Importantly, for all of these cases of experimentally determined drug synergism, a combination-specific dosage reduction was found compared to the respective single-drug treatments. Ultimately, a reduction in the range of 2- to 23-fold was obtained for EC_50_ values and 3- to 90-fold for EC_90_ values. Thus, the finding indicates that synergistic combinations between CDK7 and vCDK/pUL97 inhibitors allow for substantially reduced dosages and improved treatment efficacies compared to single-drug settings.

**Table 7:** Overview of EC_50_ and EC_90_ values of various pUL97 inhibitors (left), CDK7 inhibitor LDC4297 (right), in both cases determined for the single drugs (upper) and in combination (lower). Analysis was based on the antiviral activity measured against HCMV AD169-GFP. The x-fold dosage reduction expresses the benefit of drug combination over single-drug treatment. Each EC_50_ and EC_90_ value represents at least three independent experimental replicates based on measurements in biological duplicates.

		MBV [µM]	LDC4297 [µM]
EC_50_	single drugs	0.35 ± 0.42	0.009 ± 0.002
	in combination (100:1)	0.043	0.0004
	dosage reduction	8 × reduced	23 × reduced
EC_90_	single drugs	8.43 ± 3.68	0.124 ± 0.025
	in combination (100:1)	2.76	0.028
	dosage reduction	3 × reduced	4 × reduced
		**Ax7396** **[µM]**	**LDC4297** **[µM]**
EC_50_	single drugs	1.93 ± 0.84	0.013 ± 0.007
	in combination (100:1)	0.570 ± 0.124	0.006 ± 0.001
	dosage reduction	3 × reduced	2 × reduced
EC_90_	single drugs	8.93 ± 9.72	0.097 ± 0.047
	in combination (100:1)	2.09 ± 1.01	0.021 ± 0.010
	dosage reduction	4 × reduced	5 × reduced
		**Gö6976** **[µM]**	**LDC4297** **[µM]**
EC_50_	single drugs	1.40 ± 1.97	0.009 ± 0.002
	in combination (100:1)	0.228 ± 0.195	0.002 ± 0.002
	dosage reduction	6 × reduced	5 × reduced
EC_90_	single drugs	280.3 ± 468.6	0.147 ± 0.090
	in combination (100:1)	3.13 ± 2.69	0.031 ± 0.027
	dosage reduction	90 × reduced	5 × reduced
		**Vi7392** **[µM]**	**LDC4297** **[µM]**
EC_50_	single drugs	2.67 ± 0.36	0.016 ± 0.010
	in combination (100:1)	6 × reduced	3 × reduced
	dosage reduction	0.451 ± 0.157	0.005 ± 0.002
EC_90_	single drugs	9.08 ± 2.70	0.088 ± 0.051
	in combination (100:1)	2.16 ± 0.98	0.022 ± 0.010
	dosage reduction	4 × reduced	4 × reduced

### 2.4. Confocal Imaging-Based Analysis: The HCMV-Induced Upregulation of CDK7 Expression Levels Is Completetly Abrogated by Synergistic Drug Treatment

**Figure 3:**
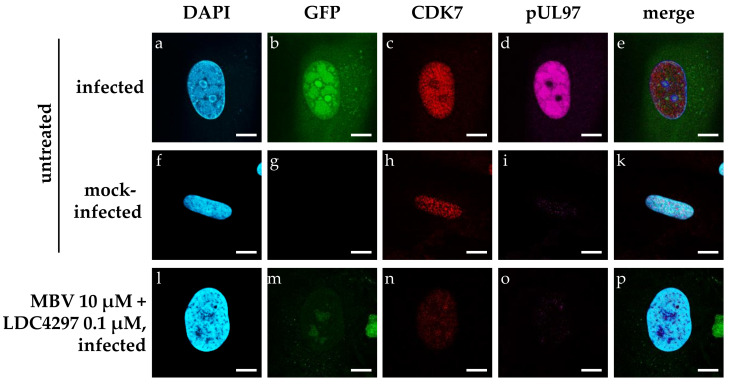
Assessment of the CDK7 and pUL97 intracellular localization and intensity via confocal microscopic analysis in mock-infected (**f**–**k**), HCMV-infected (**a**–**e**) and infected cells treated with the combination of MBV + LDC4297 (**l**–**p**). HFFs were seeded on glass cover slips und used for HCMV AG169-GFP infection (MOI of 1) or remained mock-infected. Cells were treated immediately after infection with the indicated concentrations of compounds for 7 days before fixation and staining. Scale bars represent 10 µm.

In a next step, we addressed the question of whether in vitro synergistic drug treatment exerts an influence on the localization or expression levels of target proteins. For the specific combination of LDC4297 + MBV, i.e., the drug-specific targeting of CDK7 and pUL97, we performed confocal immunofluorescence-based analysis. No drug-specific alteration of the two proteins’ intranuclear localization was observed (Figure 3c,d,h,i,n,o and Figure S4A). As expected, treatment with 10 µM MBV plus 0.1 µM LDC4297 significantly reduced infection efficiency and thereby reporter GFP expression (Figure 3b,g,m and Figure S4A,B). Interestingly, however, the CDK7 signal intensity was also decreased by drug combination treatment (Figure 3c,h,n).

**Figure 4:**
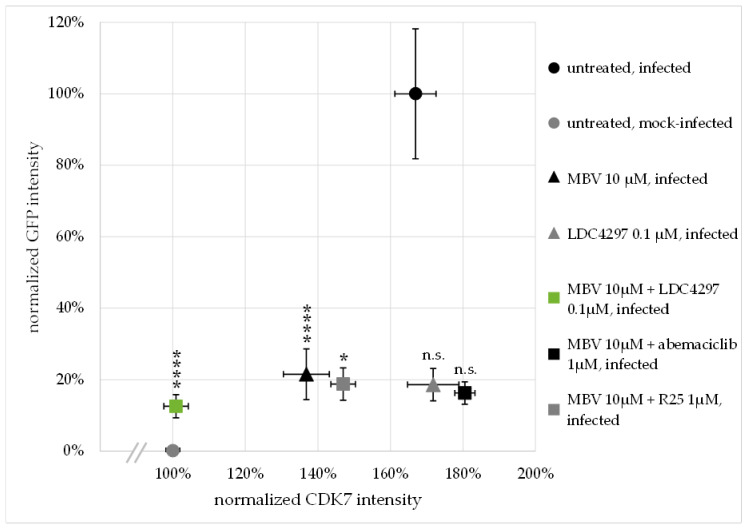
Quantitation of the CDK7 and GFP confocal immunofluorescence signals in HFFs treated with single PKIs, PKI combinations or solvent control (untreated). DAPI-corrected GFP signal was normalized with untreated, mock-infected cells as 0% and HCMV AD169-GFP-infected untreated cells as 100% (*y* axis). DAPI-corrected CDK7 signal was normalized with untreated, mock-infected cells as 100%. For each treatment condition, >90 cells were analyzed; data are shown as mean ± SEM. Statistical analysis was performed using ordinary one-way ANOVA and post-hoc Tukey’s test on CDK7 intensity values only. The most relevant synergistic drug combination, MBV + LDC4297, is highlighted in green; **** *p* ≤ 0.0001; * *p* ≤ 0.05; n.s., not significant.

To substantiate this finding, a quantitative analysis of confocal images was performed, assessing CDK7 and GFP signal intensity in >90 individual cells per treatment condition (Figure 4 and Figure S4B). For this evaluation, GFP and CDK7 signal intensity were measured via Fiji/ImageJ and normalized to DAPI signal (see 4.8 for detailed description). GFP intensity in drug-untreated, mock-infected HFFs was set as 0%; GFP intensity in untreated, HCMV-infected cells was set as 100% (Figure 4, *y* axis). CDK7 intensity in untreated, mock-infected HFFs was set as 100% (Figure 4, *x* axis), to represent the physiological state in uninfected cells. The HCMV AD169-GFP-specific reporter signal was strongly reduced below 25% by all the applied antiviral drug treatments (MBV 10 µM, LDC4297 0.1 µM, MBV 10 µM + LDC4297 0.1 µM, MBV 10 µM + R25 1 µM, MBV 10 µM + abemaciclib 1 µM). CDK7 intensity was strongly upregulated to 167% ± 6% in HCMV-infected, untreated cells (Figure 4 black circle). This HCMV-caused increase in CDK7 intensity was not reduced by treatment with LDC4297 alone (172% ± 7%, grey triangle) or the combination of MBV with the reference PKI abemaciclib (181% ± 3%, black square) and was only moderately reduced by the combination treatment of MBV with the reference PKI R25 (147% ± 3%, grey square). MBV treatment alone showed a stronger and significant reduction in CDK7 intensity (137% ± 6%, black triangle). The modulating impact of abemaciclib or R25 towards MBV, i.e., two CDK inhibitors not targeting vCDK7/pUL97, was reproducible but remains mechanistically unexplained. Importantly, however, treatment with the synergistic drug combination MBV + LDC4297 induced a drastic and highly significant reduction in CDK7 intensity, almost to mock-infected level (101% ± 3%, green square). Note that the level of GFP signal intensity, i.e., the infection efficiency, was comparable for all five treatment settings, so it is evident that this reduction in CDK7 intensity was not solely caused by the inhibition of viral replication. This finding indicated that the upregulated expression level of CDK7 in HCMV-infected cells was completely abrogated by MBV + LDC4297 treatment. The non-synergistic combinations of MBV + R25 and MBV + abemaciclib, on the other hand, showed no comparable effect on CDK7 levels while reducing viral GFP reporter expression levels to similar extent. In essence, only the MBV + LDC4297 combination treatment had a specific and strict effect on limiting the CDK7 expression level.

### 2.5. Synergy between PKIs Ax7392 and LDC4297 Is pUL97-Dependent and pUL97-Specific, as Assessed by a HCMV ΔUL97 Deletion Mutant

**Figure 5:**
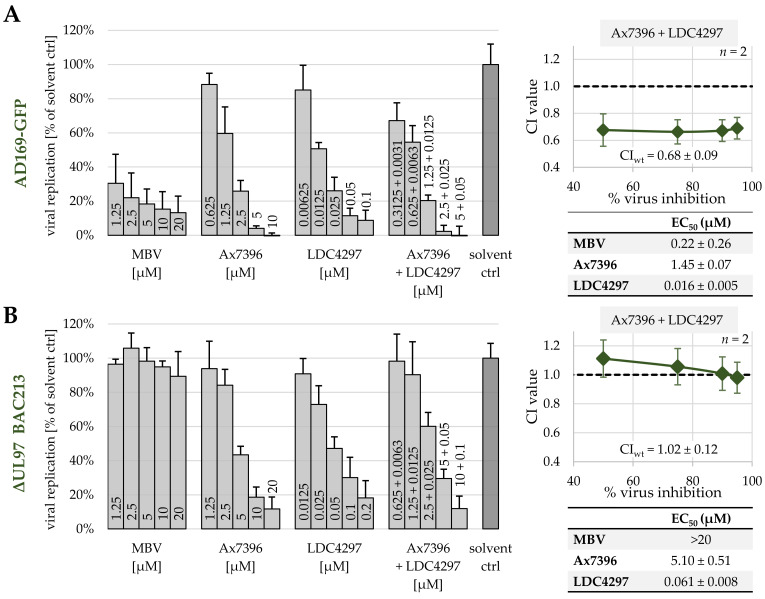
Assessment of antiviral activity of MBV, Ax7392, LDC4297 and the combination Ax7392 + LDC4297, performed in parallel with the parental HCMV AD169-GFP (**A**) and the deletion mutant HCMV ΔUL97-GFP BAC213 (**B**). Panels on the left show data from two independent GFP replication assays as mean values + SD; EC_50_ values are given in tables on the right. CI values at 50%, 75%, 90% and 95%, ± SD, as well as a weighted CI value for the combination Ax7396 + LDC4297, are given in diagrams on the right.

**Figure 6:**
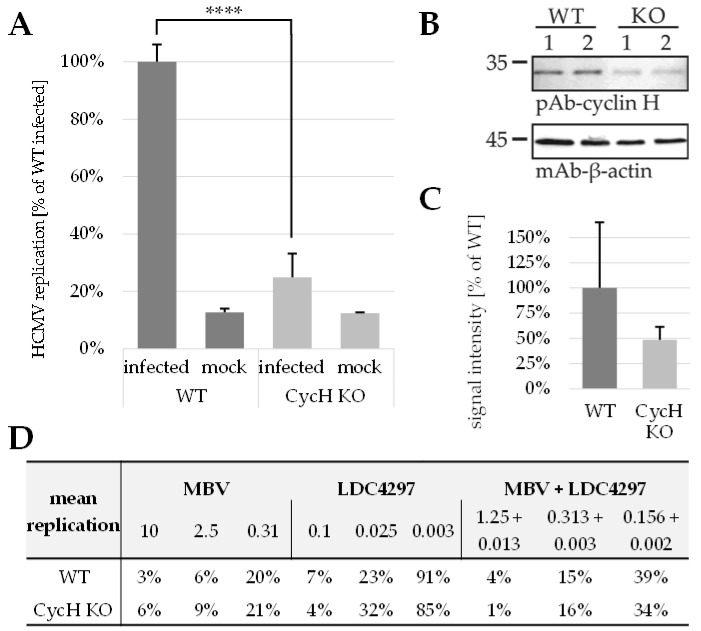
Replication of HCMV AD169-GFP is significantly reduced in HFFs with a partial cyclin H knock-out. (**A**) Comparison of HCMV replication in wild-type (WT) and cyclin H knock-out (KO) HFFs. One day after seeding, cells were treated with lentiviral supernatants for 24 h, then incubated with fresh media for 24 h, subsequently infected or mock-infected and harvested after 7 d. Data are presented as mean of biological duplicates + SD. Note that the basal signal level obtained with mock-infected samples results from excitation/emission background usually produced by the automated GFP fluorometry. Statistical analysis was performed using ordinary one-way ANOVA and posthoc Tukey’s test. (**B**) Western blot data of HFF lysates measured in A. (**C**) Densitometric analysis of Western blot bands shown in B. Data are presented as β-actin-adjusted mean densitometric values of two double-quantified bands + SD, relative to signal intensity of WT. (**D**) Mean replication values (in % solvent control) of antiviral drug treatments (in µM), compared between WT and CycH KO HFFs. WT, wild type HFFs; CycH KO, cyclin H knock-out; ****, *p* ≤ 0.0001.

**Figure 7:**
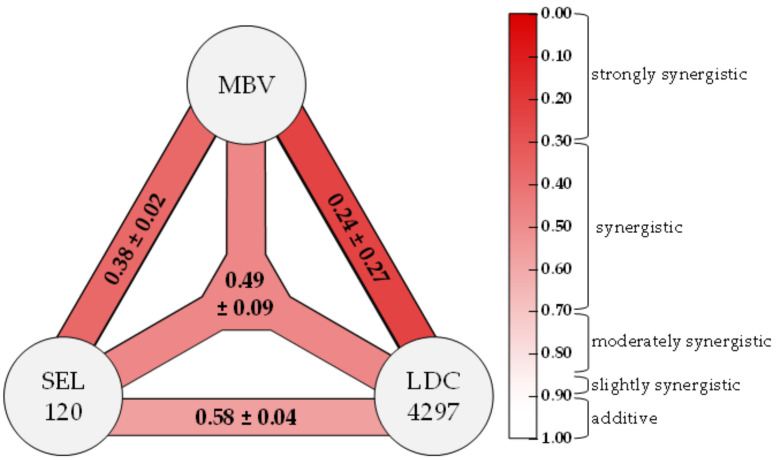
The PKIs MBV, SEL120 and LDC4297 exert synergistic antiviral activity in dual and triple combinations. Colored bars between compounds indicate the calculated strength of synergism according to color scale on the right; the three-pronged shape indicates triple synergism. Weighted CI values for each combination are given as mean values ± SD of two independent biological replicates each. Definition of synergy categories next to the color scale follows Chou et al. (2006) [32].

The recent investigations of our group, as well as the results of the present report (Figure 1, Figure 2, Figure 3, Figure 4, Figure 5, Figure 6 and Figure 7 and Figures S1–S5, Table 1, Table 2, Table 3, Table 4, Table 5 and Table 6), emphasize the high antiviral potential of compounds inhibiting the viral kinase pUL97. However, compound selectivity (i.e., mono-selectivity versus dual-/poly-selectivity) varies profoundly between inhibitors of different chemical classes [5]. For pUL97 inhibitors of the indolocarbazole class, such as Gö6976, an additional inhibition of secondary targets of the protein kinase C (PKC) group has been previously reported [31,42,43,44]. pUL97 inhibitors of the quinazoline group, such as Ax7396, Vi7392 and others, have been characterized by our studies as potent antiviral candidate compounds in vitro and in vivo [26,29,30,45,46]. For these quinazolines, reports of independent researchers likewise pointed out the role of secondary targets, in particular the epithelial growth factor receptor kinase (EGFR) [29,47]. Here, we performed additional selectivity studies for the experimental quinazoline-type inhibitor of pUL97, Vi7392, using the DiscoverX KINOMEscan Profiling system (Table S1, Figure S6). Data indicated two levels of cellular targets of Vi7392 (3 µM) on the basis of a drug competition-reduced binding at a stringency of ≤10% (S10) for the kinases CDKL1 and PIP5K2C and, in addition, at a lower stringency of ≤35% (S35) for the kinases CDKL1, PIP5K2C, EGFR, MAPK2K5, MKNK2 and PRKG2 (note that cancer-specific kinase mutants of EGFR and FLT3, which do not have a known relevance for HCMV infection, were not further considered in this context). Thus, the two host kinases CDKL1 and PIP5K2C (and possibly additional kinases for level S35) have to be considered as secondary targets of Vi7392. This finding clearly supports the poly-selective nature of this quinazoline-class kinase inhibitor.

Building on these findings, another assessment of PKI synergy effects was performed for the related quinazoline Ax7396, this time using the ORF-UL97-deleted HCMV of the recombinant strain AD169-GFP, termed ΔUL97-GFP BAC213 (Marschall et al., 2005 [48]; Figure 3). For comparison, the parental HCMV AD169-GFP was used to illustrate the antiviral activity of pUL97 inhibitors MBV and Ax7396, the CDK7 inhibitor LDC4297 and the combination setting Ax7396 + LDC4297. All treatments exhibited strong antiviral activity towards HCMV AD169-GFP, especially the combination Ax7396 + LDC4297, confirming its synergistic effect (CI_wt_ = 0.68; Figure 3a, right panel). Next, identical infection settings were applied for HCMV ΔUL97-GFP BAC213 (Figure 3b). In this situation, MBV visibly lost its anti-HCMV activity, thus confirming earlier statements of the high pUL97-directed selectivity of this drug. In contrast to MBV, however, Ax7396 still exerted a measurable level of anti-HCMV activity, indicating a lack of pUL97 selectivity. The antiviral activity of Ax7396 towards the ΔUL97 virus was obviously based on the inhibition of secondary, virus-supporting cellular target kinases. Importantly, the combination treatment Ax7396 + LDC4297 did not exhibit a synergistic effect in a HCMV ΔUL97-GFP BAC213 infection setting, but instead exhibited an additive interaction (CI_wt_ = 1.02; Figure 3b, right panel). It should also be mentioned in this context that earlier results excluded a secondary activity of CDK7 inhibitor LDC4297 towards viral pUL97 [21]. This result shows that the synergism between Ax7392 and LDC4297 is pUL97-dependent and pUL97-specific and thereby underlines the importance of pUL97 as a combinatorial drug target. This strengthens our hypothesis of a direct mechanistic interaction between viral kinase pUL97 and host kinase CDK7.

### 2.6. PKI Synergy Specifically Reinvestigated Using Host Cell Populations with Cyclin H Knock-Out

The very strong antiviral activity of CDK7 inhibitors prompted us to perform additional experimentation using cyclin knock-out cell populations. Specifically, a cyclin H knock-out was expected to provide a cellular environment lacking functional CDK7–cyclin H complexes, which might also prevent the target-specific antiviral effect of CDK7 inhibitor LDC4297. To this end, we generated lentiviral constructs expressing the CRISPR/Cas module for a guide RNA-directed knock-out of cyclin H. Interestingly, a selection of HFF knock-out populations by the transduction-specific antibiotic resistance marker (zeocin) did not lead to a stable reduction in cyclin H expression. While a clear decrease in cyclin H expression levels was detected by western blot (Wb) using total cellular lysates during the first cell passages of selection, this reduction was found to be reversed in later passages (data not shown). Apparently, the cyclin H knock-out was subject to counterselection through cells lacking the knock-out but carrying the selection marker, so that the resulting populations returned to express almost wild-type levels of cyclin H. This process underlines the importance of cyclin H expression in primary human fibroblasts and its selection pressure. Consequentially, we performed a transient knock-out (Figure 6A–D), in which the transduced cells were not passaged under antibiotic selection but were cultivated in 12-well plates, directly treated with lentiviral constructs and immediately used for HCMV infection (Figure 6A). Fortunately, the levels of cyclin H could be reduced substantially in this approach (2.06-fold mean reduction to 48.6% of wild-type level; Figure 6B,C). Notably, this partial defect in cyclin H expression significantly reduced the rate of infectivity with HCMV AD169-GFP (4.03-fold mean reduction to 24.8% of infected HFF wild-type level; Figure 6A). When utilizing this limited percentage of virus-infected cells for the analysis of antiviral drug activity, no marked difference was seen between cyclin H knock-out and wild-type cells. The effects of antiviral treatment were very similar for the three drug settings analyzed, i.e., LDC4297 alone, MBV alone and MBV + LDC4297 in combination (Figure 6D and Figure S5). This finding indicates that the approach with transient cyclin H knock-out (that basically reduced HCMV infection levels) did not alter the sensitivity of HCMV replication towards these drugs, which is especially surprising in the case of LDC4297. As this inhibitor shows a high selectivity for CDK7 [21,49], the sustained antiviral activity of the drug under cyclin H knock-out may be explained by two effects. Firstly, the level of partial knock-out under these transient conditions may not have been sufficient to prevent the activating role of CDK7–cyclin H for HCMV replication. This appears unlikely, however, since overall virus infection was found very low in these cells. An alternative explanation may be a CDK7-directed, complementing effect by related cyclins under cyclin H knock-out, such as cyclin T1 or others. CDK7 has been described as single cyclin binding [50,51], but this characteristic has not been analyzed for the regulatory role of CDK7 towards HCMV replication. It appears possible that in the case of the HCMV-supportive function of CDK7, this kinase might switch to multiple cyclin binding properties, in particular under the situation of experimental cyclin knock-out (Marschall, Schütz et al., unpublished results). It should be mentioned in this regard that the viral CDK ortholog pUL97 is also capable of multiple cyclin binding, including the types H, T1 and B1 [5,52,53].

### 2.7. Triple Drug Combination Treatment Additionally Optimizes the Synergistic Potency of Anti-HCMV Activity of PKIs

The efficacy of combinatorial anti-HCMV drug treatments in vitro was further assessed, specifically taking into account that in clinical antiviral settings, such as the anti-retroviral/AIDS therapy against HIV-1 infections, a treatment with triple drug combinations is preferentially applied [54,55]. Thus, we addressed the question whether the use of three PKIs directed against three different target kinases may likewise lead to a clear, algorithm-defined synergistic anti-HCMV effect. This approach was intended to establish an improved strategy for the comparison between dual and ternary targeting synergisms using the Loewe additivity fixed-dose assay. To this end, we analyzed the dual and triple combinations of MBV, LDC4297 and SEL120 in the HCMV GFP-based replication assay (Figure 7). These data indicated drug synergism for all combinations analyzed. Strongly synergistic anti-HCMV activity was indicated for MBV + LDC4297 (CI_wt_ of 0.24 ± 0.27), as reported earlier [1]. In addition, synergistic interactions were also identified for all further combinations, i.e., dual, MBV + SEL120 (0.38 ± 0.02) and SEL120 + LDC4297 (0.58 ± 0.04) as well as the triple treatment of MBV + LDC4297 + SEL120 (0.49 ± 0.09). In particular, the triple drug combination may be relevant for the further development of treatment strategies, as it is based on a combination of direct antiviral (vCDK/pUL97) and host-directed targets (CDK7 and CDK8). This situation may help, on the one hand, to strongly increase viral resistance barriers and, on the other hand, to allow for a substantial reduction in the dosage of the individual drug components. Thus, the result strongly supports our notion that viral and cellular kinases represent highly attractive targets for synergistic drug combinations.

### 2.8. Conclusions

The data of the present report support the following conclusions: Anti-HCMV drug synergy is exhibited by pairs of kinase inhibitors, belonging to various chemical classes, directed to viral pUL97 and host CDK7. These drug combinations do not induce an amplification of cytotoxicity. Specifically, synergistic drug combinations lead to a reduction in drug dosage levels when compared to monotreatments. On the molecular level, the expression of the target protein CDK7 is significantly down-modulated under the conditions of CDK7-/pUL97-directed synergistic drug treatment, and the functional importance of these two cyclin H-associated target kinases is specifically measurable in HCMV infection settings using ORF-UL97-deleted virus or cellular cyclin knock-out. Moreover, new combinations of HCMV-specific drug synergy can also be achieved by host-directed strategies using PKIs against CDK2, CDK7, CDK8 and/or CDK9. Finally, a first example of triple drug combination provides evidence for the extraordinary potential of this PKI-based synergy approach.

## 3. Discussion

The previous reports of our group and other researchers underlined the multifaceted functional roles of CDK–cyclin complexes in herpesviral replication. Specifically, distinct CDKs and the cytomegaloviral CDK ortholog pUL97 have been validated as promising targets of novel antiviral strategies. Recent findings demonstrated that combinatorial drug treatments with clinically relevant PKIs can exert promising anti-HCMV activity. This aspect was initially underlined by the identification of the true synergistic effect of drug combinations directed against human CDK7 and viral pUL97. Here, we strongly substantiated these findings with a broader panel of compounds showing highly attractive antiviral efficacies and MoA. In essence, we demonstrated that drug synergy was exerted by pairs of PKIs from various chemical classes directed to pUL97 and CDK7 in the absence of putative amplification of cytotoxicity. These data revealed a substantial reduction in drug dosages, thereby further illustrating the benefit of drug combination over single-drug treatment. Moreover, drug synergism was also achieved through specific combinations of two host-directed PKIs (directed against CDK2, CDK7, CDK8 and/or CDK9). Interestingly, the new data also demonstrated that the HCMV-induced upregulation of CDK7 expression levels was completely abrogated by synergistic drug treatment. Another mechanistic aspect of these synergistic drug combinations was illustrated by the use of a HCMV ΔUL97 deletion mutant and cyclin H knock-out cells, confirming the quality of this targeting strategy. Finally, we provided the first evidence for a triple PKI combination possessing synergistic potency in terms of anti-HCMV activity.

During the last two decades, a number of specific pharmacological CDK inhibitors have been developed and approved for cancer treatment. The further development of these CDK-directed PKIs for the treatment of viral infections may represent a novel effective therapeutic strategy to combat old and emerging viruses [4]. In general, viruses rely on the host cell for resources to create a favorable environment of viral replication. To this end, they use a variety of mechanisms to reprogram and control cellular activities, with the manipulation of the host cell cycle being one of the most frequent points of action. This complex and interactive way of controlling kinase-driven cell cycle machinery and signaling pathways introduces many opportunities for viral manipulation [4,56]. As far as the function of CDK7 in cell cycle regulation is concerned, the trimeric complex CDK7–cyclin H-MAT1, commonly called the CDK-activating kinase (CAK) complex [57], fulfils a CDK-activating step. Moreover, CDK7 represents a component of the general transcription factor TFIIH and is involved in the phosphorylation of serine residues of the RNA polymerase II C-terminal domain (RNAP II-CTD). Interestingly, we and others showed that phosphorylation levels within the RNAP II-CTD are unaffected when CDK7 activity is inhibited in fibroblasts [21]. This finding suggests that CDK7 might not be essential for global RNAP II-driven transcription but that its functional deficiency in RNAP II regulation may be compensated by other kinase activities. In contrast, the loss of CDK7 activity is critical for cell cycle control since it leads to a lack of normal CAK function and induces cell cycle arrest. Interestingly, the mode of antiviral activity of the CDK7-specific inhibitor LDC4297 already manifests at the immediate early (IE) phase of HCMV replication. Since the progression of lytic HCMV replication is strictly dependent on IE gene expression, this specific drug activity is translated into a drastic limitation of all downstream viral replication events [21].

When speculating about the regulatory basis of synergistic antiviral drug interactions, one might think about potentially special features of drug–target interaction. It should be stressed, however, that the drugs applied in this study represent classical ATP-competitive kinase inhibitors, and thus it appears unlikely that the mode of inhibition and the drug binding characteristics may reveal unexpected details. It is much more probable that the emergence of drug synergy stems from the interactive relation between the specific target kinases. Thus, antiviral drug synergy is most probably not determined at the level of drug–target interaction, but at the level of target–target interaction, such as a functional cross-talk between the target kinases. Basically, we suggest three theoretical MoA concepts of kinase interactive relationships associated with drug synergy: (i) two drug-targeted activities are both crucially important, or even absolutely essential, for efficient virus replication, (ii) two drug-targeted activities are functionally related, for instance through an overlapping substrate spectrum, or (iii) two drug-targeted activities are dependent on each other, for instance through mutually activating cross-talk or similar feedback effects. In all these cases, the co-inhibition of two kinase activities, through the respective drug combination treatment, may result in the described synergistic effects leading to strongly increased antiviral efficacy.

Concerning the specific case of antiviral activity exerted by CDK7 inhibitors, we state that at least three mechanistic aspects may have a combined impact on their activities, as based on our previous analyses [1,5,21,24,25]. Firstly, the transcription-directed regulatory role of CDK7 activity is considered to be required for efficient HCMV replication. Secondly, its cell cycle-specific CAK master activity is seen to exert an even stronger impact. Thirdly, and possibly more important than previously expected, the direct phosphorylation of viral proteins through CDK-cyclin complexes (M.M. et al., unpublished data) could have an importance for substrate protein activity and the efficiency of lytic viral replication. It should be mentioned that we previously demonstrated the formation of ternary complexes between CDK7, cyclin H and viral pUL97 [53].

Given this background knowledge, the regulatory role of CDK7 activity for HCMV replication appears to be crucial and should thus be accessible to modern antiviral strategies. In particular, the combined drug targeting of CDK and vCDK/pUL97 activities may enable untapped therapeutic possibilities. It should be emphasized again that, very recently, MBV has been approved as the first kinase inhibitor in the entire field of antiviral therapy, and that further kinase inhibitors like LDC4297 and related analogs are presently being investigated at the preclinical/clinical level. In order to validate the specific benefit of the PKI combination strategy in antiviral treatments, a proof-of-concept has to be achieved in an animal model. So far, the examples of strict algorithm-based calculations of drug synergy through the use of data derived from animal experiments have been rare [58], and thus we aim to demonstrate our described synergistic PKI combinations in vivo. At present, we are further developing the previously established MCMV/mouse infection model [1,24,59,60], so that an in vivo proof-of-concept might lift our synergy strategy to the next level of antiviral research. With our combined findings, this study highlights the potential of therapeutic drug combinations of approved, developmental and preclinical PKIs for broadening the scope of future anti-HCMV therapy.

## 4. Materials and Methods

### 4.1. Cells and Viruses

Primary human foreskin fibroblasts (HFFs, derived from clinical samples, Children’s Hospital, Erlangen, Germany) were grown in Eagle’s minimal essential medium (MEM) supplemented with 1 × GlutaMAX^TM^ (both Thermo Fisher Scientific, Waltham, MA, USA), 10 μg/mL gentamicin and 10% fetal bovine serum (FBS, Capricorn, Ebsdorfergrund, Germany). Human embryonic kidney epithelial cells (293Ts, ATCC, Manassas, VA, USA) were grown in Dulbecco’s modified Eagle medium (DMEM) supplemented with 1 × GlutaMAX^TM^ (both Thermo Fisher Scientific, Waltham, MA, USA), 10 μg/mL gentamicin and 10% FBS (Capricorn, Ebsdorfergrund, Germany). Cultured cells were maintained at 37 °C, 5% CO_2_ and 80% humidity. All cell culture was regularly monitored for absence of mycoplasma contamination (Lonza™ Mycoalert™, Thermo Fisher Scientific, Waltham, MA, USA). Recombinant HCMV AD169 expressing green fluorescent protein (AD169-GFP, [61]) and recombinant ORF-UL97-deleted HCMV AD169 expressing green fluorescent protein (ΔUL97-GFP BAC213 [48]) were used for in vitro replication assays.

### 4.2. Antiviral Compounds

Antiviral drugs were obtained from the following sources: Calbiochem, Darmstadt, Germany (CDK2 Inh II, Gö6976); GPC Biotech AG, Martinsried, Germany (R25); Lead Discovery Center GmbH, Dortmund, Germany (LDC4297); MedChemExpress, Monmouth Junction, NJ, USA (abemaciclib, AZD4573, CVT-313, CYC065, dinaciclib, maribavir, riviciclib, samuraciclib SEL120, SY1365); Tocris, Wiesbaden-Nordenstadt, Germany (SNS032, THAL-SNS032); Vichem Kft, Budapest, Hungary (Ax7396, Vi7392). Stock aliquots were prepared in sterile DMSO (Sigma Aldrich, St. Louis, MO, USA) and stored at −20 °C.

### 4.3. Antibodies

The following antibodies were used in this study: monoclonal mouse anti-CDK7 (Sc-56284; Santa Cruz Biotechnology, Dallas, TX, USA), polyclonal rabbit anti-UL97 (courtesy of Dr. Donald M. Coen, Boston, MA, USA), Alexa 555 goat anti-rabbit (A21429), Alexa 488 goat anti-mouse (A11029, both Thermo Fisher Scientific, Waltham, MA, USA).

### 4.4. Determination of Cell Viability by Neutral Red Uptake Assay

Drug induced cytotoxicity in HFFs was measured as a reduction of cell viability determined by neutral red uptake assay (NRA), as described previously [62,63]. Briefly, HFFs treated with compounds for 7 d were incubated with a final concentration of 40 μg/mL neutral red (Sigma Aldrich, St. Louis, MO, USA) for 3 h. Incorporated neutral red was released from the cells by incubation with destaining solution (50% ethanol, 49% H_2_O, 1% acetic acid) and subsequently quantitated in a microplate reader (PerkinElmer, Waltham, MA, USA) by fluorescence measurement using 560/630 nm for excitation/emission, respectively.

### 4.5. Drug Interaction Assessment via Bliss Independence Checkerboard Assay Adapted to HCMV-GFP In Vitro Infection

Bliss-based drug interaction was assessed using an adapted protocol of the HCMV GFP-based replication assay described previously [1,21,61]. HFFs were seeded at app. 1.2 × 10^4^ cells/well in 96-well culture plates (three plates per assay) and infected on the following day with HCMV AD169-GFP [61] in a dilution resulting in 25% GFP-positive cells at 7 d p.i. (i.e., 1 × TCID_25_^7d^) or remained mock-infected. After a virus adsorption phase of 90 min, the inoculum was replaced by medium supplemented with a matrix of drug combinations in different concentration ratios, solvent control or medium for mock-infected wells. Standard protocol tested a matrix of 8 × 8 concentrations, beginning with ~8 × EC_50_ and 1:2 dilutions. All infections were performed in biological triplicates. Cells were lysed by the addition of 100 µL lysis buffer/well 7 d p.i., and cell suspensions were mixed and transferred to another 96-well plate. Centrifugation was performed at 3000 rpm for 15 min and clear lysates were subjected to automated GFP quantitation in a Victor X4 microplate reader (PerkinElmer, Waltham, MA, USA). Measured values were entered into the MacSynergy II software [64] and results were presented as surface graphs illustrating synergy volume with a 95% confidence interval over the three biological replicates.

### 4.6. Drug Interaction Assessment via Loewe Additivity Fixed-Dose Assay Adapted to HCMV-GFP In Vitro Infection

Loewe additivity was assessed using an adapted protocol of the HCMV GFP-based replication assay [21,61] described previously [1]. HFFs were seeded at app. 1.6 × 10^5^ cells/well in 12-well culture plates (four plates per assay) and infected on the following day with HCMV AD169-GFP [61] in a dilution resulting in 25% GFP-positive cells at 7 d p.i. (i.e., 1× TCID_25_^7d^). After virus adsorption, the inoculum was replaced by medium supplemented with single compound, compound combination or solvent control. Standard protocol comprised a serial dilution of six concentrations/single compound, starting with app. 4–8 × EC_50_, and a 1:2 serial dilution of eight concentrations of the combination, starting with a combination of half of the highest concentrations of both single dilution series. All infections were performed in biological duplicates. Cells were lysed by the addition of 200 µL lysis buffer/well 7 d p.i., and cell suspensions were mixed and transferred to a 96-well plate. Centrifugation was performed at 3000 rpm for 15 min and clear lysates were subjected to automated GFP quantitation in a Victor X4 microplate reader (PerkinElmer, Waltham, MA, USA). Antiviral efficacy (mean of duplicate measurement of biological duplicates) was expressed as the percentage of solvent control and entered into the CompuSyn software (Version 1.0 [65]; ComboSyn, Inc., Paramus, NJ, USA). Only experiments with an r value > 0.90 and EC_50_ values close to previously determined concentrations were accepted.

### 4.7. Indirect Immunofluorescence Assay Utilizing Confocal Laser-Scanning Microscopy

For immunofluorescence detection, app. 2 × 10^5^ HFFs/well were grown on coverslips in six-well culture plates and used for infection with HCMV AD169-GFP [61] at an MOI of 1 or remained mock-infected. After virus adsorption, the inoculum was replaced by medium supplemented with single compound, compound combination or solvent control. At 7 d p.i., cells were fixed with 10% formalin solution (Sigma Aldrich, St. Louis, MO, USA; 10 min, room temperature) and permeabilized by incubation with 0.2% Triton X-100 solution (Roth, Karlsruhe, Germany; 20 min, 4 °C). Nonspecific staining was blocked by incubation with 2 mg/mL human γ-globulin Cohn fraction II, (Merck, Darmstadt, Germany; 30 min, 37 °C). Indirect immunofluorescence staining was performed by incubation with primary antibody diluted in PBS (60 min, 37 °C), washing and subsequent incubation with diluted dye-conjugated secondary antibody (30 min, 37 °C). After further washing steps, cells were mounted with VECTASHIELD^®^ mounting medium with DAPI (Vector Laboratories, Burlingame, CA, USA), before glass coverslips were sealed using nail polish. Confocal laser-scanning microscopy was performed with a TCS SP5 microscope using a 63× HCX PL APO CS oil immersion objective lens (Leica Microsystems, Mannheim, Germany). Images were processed using the LAS AF software (version 2.6.0 build 7266; Leica Microsystems, Mannheim, Germany) and edited with Photoshop CS5 (Adobe, San José, CA, USA).

### 4.8. Quantitation of Intracellular Immunofluorescence Signals

Intensity of GFP and CDK7 signals of cells, fixed and stained as described above, was measured utilizing Fiji/ImageJ (version 1.52p, [66]). For this purpose, confocal microscopy images taken under identical setting conditions were converted to 16-bit format. Subsequently, the DAPI channel image was converted to binary and outlines of nuclei were used as a template to measure GFP/CDK7 intensity in the respective channels (tool: *Analyze particles*; setting: *Size* (*inch*^2^) = 0.50 − *Infinity*). Outlines of measured objects were visually monitored in order to exclude artefacts or multiple nuclei measured as one. The GFP/CDK7 intensity was further normalized to the DAPI intensity of the respective nucleus in order to exclude z plane variation effects.

### 4.9. Generation of Lentiviral Transfer Constructs

Cyclin H-specific guide RNAs (gRNAs) were designed using the benchling.com software (Benchling, San Francisco, CA, USA) and the scoring method of Hsu et al., 2013 [67]. gRNAs were directed to cyclin H exons 1–5 and had on-target scores between 52.8 and 73.4 out of 100 and off-target scores between 43.1 and 82.9. The following gRNAs were used: Cyclin H A: 5′ GCCGCTTCTGACTACTGTTG 3′; Cyclin H B: 5′ GTCCAAGAGGACTCTCCCGG 3′; Cyclin H C: 5′ ATTCTCCAATATGGGATAGC 3′; Cyclin H D: 5′ ACAGTAGTCAGAAGCGGCAC 3′; Cyclin H E: 5′ CCGGGAGAGTCCTCTTGGAC 3‘; Cyclin H F: 5′ CAGTAATGGAATATCACCCC 3′. Cyclin H-specific CRISPR/Cas9 vectors (pLentiCRIPSR-V2) were generated according to the standard Zhang laboratory protocol [67,68,69]. 293T cells were transfected with pLentiCRISPR-V2 together with a packaging plasmid mix coding for HIV-1 gag/pol/rev and the envelope protein G of vesicular stomatitis virus (VSV-G) using Lipofectamine 2000 (Invitrogen, Karlsruhe, Germany). Lentiviral supernatants were harvested 48 h post-transfection, centrifuged for 5 min at 2000 rpm to remove cell debris, filtered and stored in aliquots at −80 °C.

### 4.10. Transient Cyclin H Knock-Out of HFFs

To achieve a transient knock-out of cyclin H, app. 1.5 × 10^5^ HFFs/well were grown in 12-well culture plates and, starting the following day, incubated for 24 h with 0.5 mL of lentiviral supernatant (a mixture containing all six gRNAs described in 4.9) supplemented with 0.5 mL MEM and 7.5 µg/mL polybrene (Sigma Aldrich, St. Louis, MO, USA) per well. Virus supernatant was replaced by fresh media 24 h later and cells were used for infection experiments (see 4.5 and 4.6) on the following day.

## Figures and Tables

**Table 1:** Single treatments: anti-HCMV-specific EC_50_, HFF-specific CC_50_ and SI values of selected PKIs.

Target	Compound	EC_50_ ^a^	CC_50_ ^b^	SI ^c^
vCDK/ pUL97	maribavir (MBV)	0.35 ± 0.42	>100	>250
	Gö6976	1.40 ± 1.99	>100	>70
	Ax7396	1.93 ± 0.84	>100	>50
	Vi7392	2.67 ± 0.36	57.8 ± 13.9	22
CDK2	CDK2 Inh II	6.33 ± 2.80	>100	>16
CDK7	LDC4297	0.009 ± 0.002	>10	>1000
CDK8	SEL120	0.079 ± 0.001	5.90 ± 3.85	75
CDK9	THAL-SNS032	0.025 ± 0.002	0.14 ± 0.06	6
CDK2	CVT-313	nd	5.57 ± 0.05	nd
CDK7	SY1365	nd	<0.1	nd
CDK7	samuraciclib	nd	0.50 ± 0.21	nd
CDK9	AZD4573	nd	0.35 ± 0.09	nd
CDK1/2/5/9	dinaciclib	nd	<0.1	nd
CDK1/4/9	riviciclib	nd	1.6 ± 0.06	nd
CDK2/9	CYC065	nd	<0.1	nd

^a^ Half-maximal efficacy (EC_50_) against HCMV AD169-GFP was determined via GFP-based replication assay; these EC_50_ values represent the mean of at least two biological replicates each ± SD (data not shown). ^b^ Half-maximal cytotoxicity on mock-infected primary human forreskin fibroblasts (HFFs) was determined via neutral red assay (NRA); these CC_50_ values represent the mean ± SD of at least two biological replicates each (Figures S2 and S3). ^c^ Selectivity index was calculated as CC_50_/EC_50_; nd, not determined.

**Table 2:** Combination treatments: anti-HCMV-specific EC_50_, HFF-specific CC_50_ and SI values of selected PKI combinations.

Compound A	Compound B		Ratio	EC_50_ ^a^	CC_50_ ^b^	SI ^c^
MBV	LDC4297		100:1	0.06 ± 0.08	>50	>800
Vi7392	LDC4297		100:1	0.51 ± 0.44	>50	>90
Gö6976	LDC4297		100:1	0.23 ± 0.20	19.4 ± 7.7	84
Ax7396	LDC4297		100:1	0.58 ± 0.13	25.8 ± 2.7	44
SEL120	LDC4297		10:1	0.04 ± 0.02	0.14 ± 0.09	4
MBV	THAL-SNS032		100:1	0.26 ± 0.16	11.2 ± 0.90	43
SEL120	THAL-SNS032		50:1	0.19 ± 0.12	0.47 ± 0.04	2
MBV	CDK2 Inh II		1:1	0.01 ± 0.00	>50	>5000
LDC4297	CDK2 Inh II		1:100	3.3 ± 2.7	>50	>15
SEL120	CDK2 Inh II		1:10	0.10 ± 0.06	>50	>500
MBV	SEL120		10:1	0.15 ± 0.07	7.7 ± 1.2	51
**Compound A**	**Compound B**	**Compound C**	**Ratio**	**EC_50_ ^a^**	**CC_50_ ^b^**	**SI ^c^**
MBV	LDC4297	SEL120	100:1:10	0.18 ± 0.01	48.7 ± 0.71	270

^a^ Half-maximal efficacy against HCMV AD169-GFP was determined via GFP-based replication assay; these EC_50_ values represent the mean of at least two biological replicates each ± SD (data not shown). ^b^ Half-maximal cytotoxicity on mock-infected HFFs was determined via NRA; these CC_50_ values represent the mean of at least two biological replicates each ± SD (Figure S1). ^c^ Selectivity index was calculated as CC_50_/EC_50_.

## Data Availability

Not applicable.

## Supplementary Materials

Supplementary Materials are available online at www.mdpi.com/article/10.3390/ijms23052493/s1.

## Abbreviations

AIDS	acquired immunodeficiency syndrome
app.	approximately
CAK	CDK-activating kinase
cCMV	congenital cytomegalovirus infection
CDK	cyclin-dependent kinase
CDV	cidofovir
CI	combination index
CI_wt_	weighted combination index
CTD	C-terminal domain
CycH	cyclin H
d	day(s)
DAA	direct-acting antiviral(s)
DMEM	Dulbecco’s modified Eagle’s medium
DMSO	dimethyl sulfoxide
EGFR	epithelial growth factor receptor kinase
FBS	fetal bovine serum
FOS	foscarnet
GCV	ganciclovir
GFP	green fluorescent protein
gRNA	guide RNA
HCMV	human cytomegalovirus
HDA	host-directed antiviral(s)
HFF	human foreskin fibroblast
HIV	human immunodeficiency virus
IE	immediate early
KO	knock-out
LMV	letermovir
MBV	maribavir
MEM	Eagle’s minimal essential medium
min	minute(s)
MoA	mode of action
MOI	multiplicity of infection
NRA	neutral red assay
ORF	open reading frame
p.i.	post-infection
PBS	phosphate buffered saline
PKC	protein kinase C
PKI	pharmaceutical kinase inhibitor
RNAP	RNA polymerase
rpm	rotations per minute
SD	standard deviation
SEM	standard error of the mean
SV	synergy volume(s)
TCID	tissue culture infectious dose
VGCV	valganciclovir
Wb	western blot
WT	wild-type
