# Supplementary material for: Cyclin-Dependent Kinases (CDKs) and the Human Cytomegalovirus-Encoded CDK Ortholog pUL97 Represent Highly Attractive Targets for Synergistic Drug Combinations
Source: Int J Mol Sci. 2022 Feb 24;23(5):2493. doi: 10.3390/ijms23052493 (PMC8910733; doi:10.3390/ijms23052493)
Supplement: Supplementary file 1 [file ijms-23-02493-s001.zip › ijms-1594508-supplementary.pdf]

# Supplementary Material

## Cyclin-dependent kinases (CDKs) and the human cytomegalovirus-encoded CDK ortholog pUL97 represent highly attractive targets for synergistic drug combinations

**Markus Wild[1], Friedrich Hahn[1], Nadine Brückner[1], Martin Schütz[1], Christina Wangen[1], Sabrina Wagner[1], Mona Sommerer[1], Stefan Strobl[2,3], Manfred Marschall[1,*]**

[1] Institute for Clinical and Molecular Virology, Friedrich-Alexander University of Erlangen-Nürnberg (FAU), Schlossgarten 4, 91054 Erlangen, Germany; markus.wild@uk-erlangen.de, friedrich.hahn@uk-erlangen.de, nadine.brueckner@fau.de, martin.schuetz@uk-erlangen.de, christina.wangen@uk-erlangen.de, sabrina.wagner@uk-erlangen.de, monas.sommerer@fau.de, manfred.marschall@fau.de

[2] 4SC AG/4SC Discovery GmbH, Fraunhoferstraße 22, 82152 Planegg-Martinsried, Germany; stefan.strobl@biontech.de

[3] Present address: BioNTech SE, Am Klopferspitz 19a, 82152 Planegg-Martinsried, Germany

* Correspondence: manfred.marschall@fau.de, Tel.: ++49 9131 8526089

# Supplementary Figures

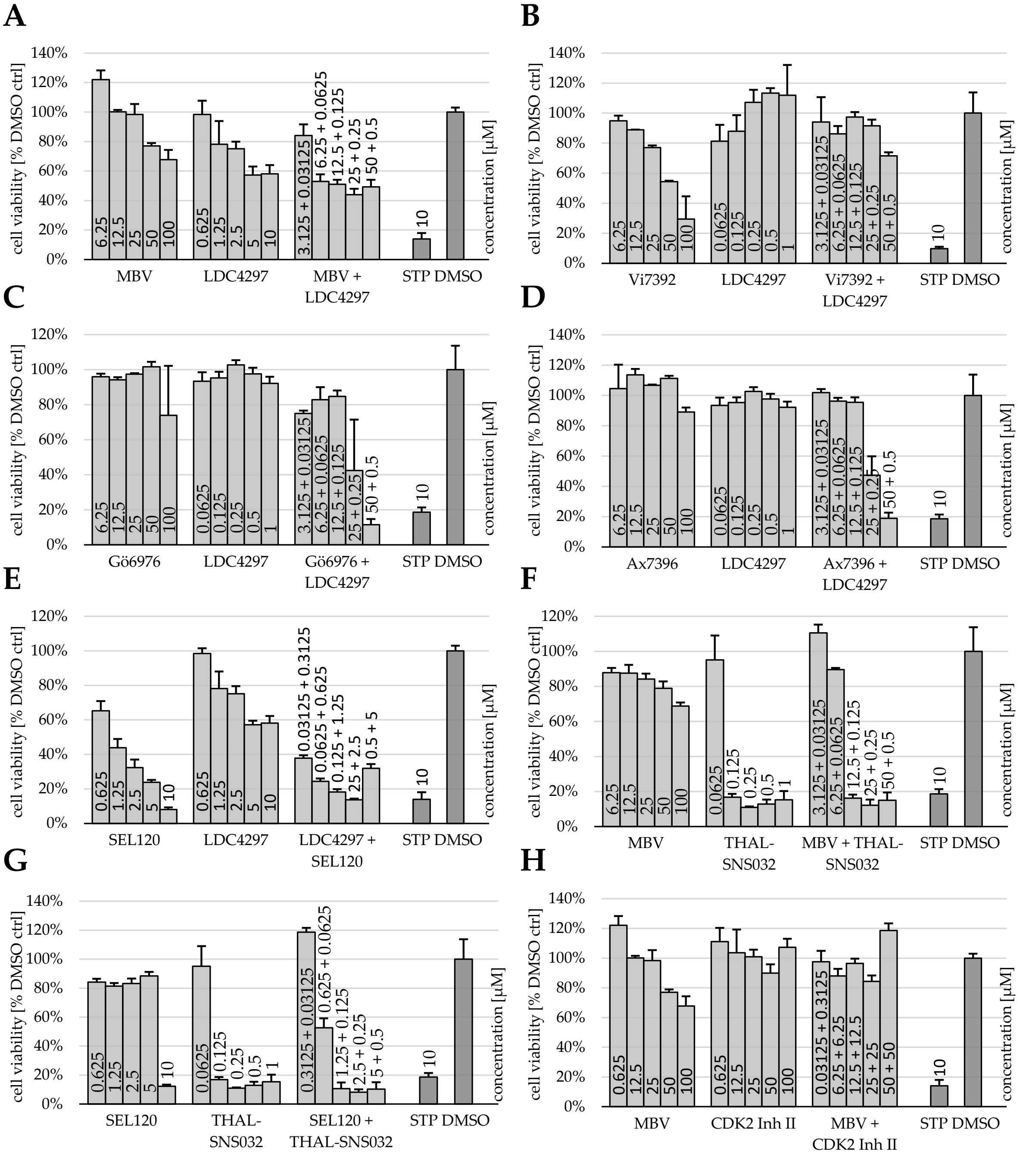

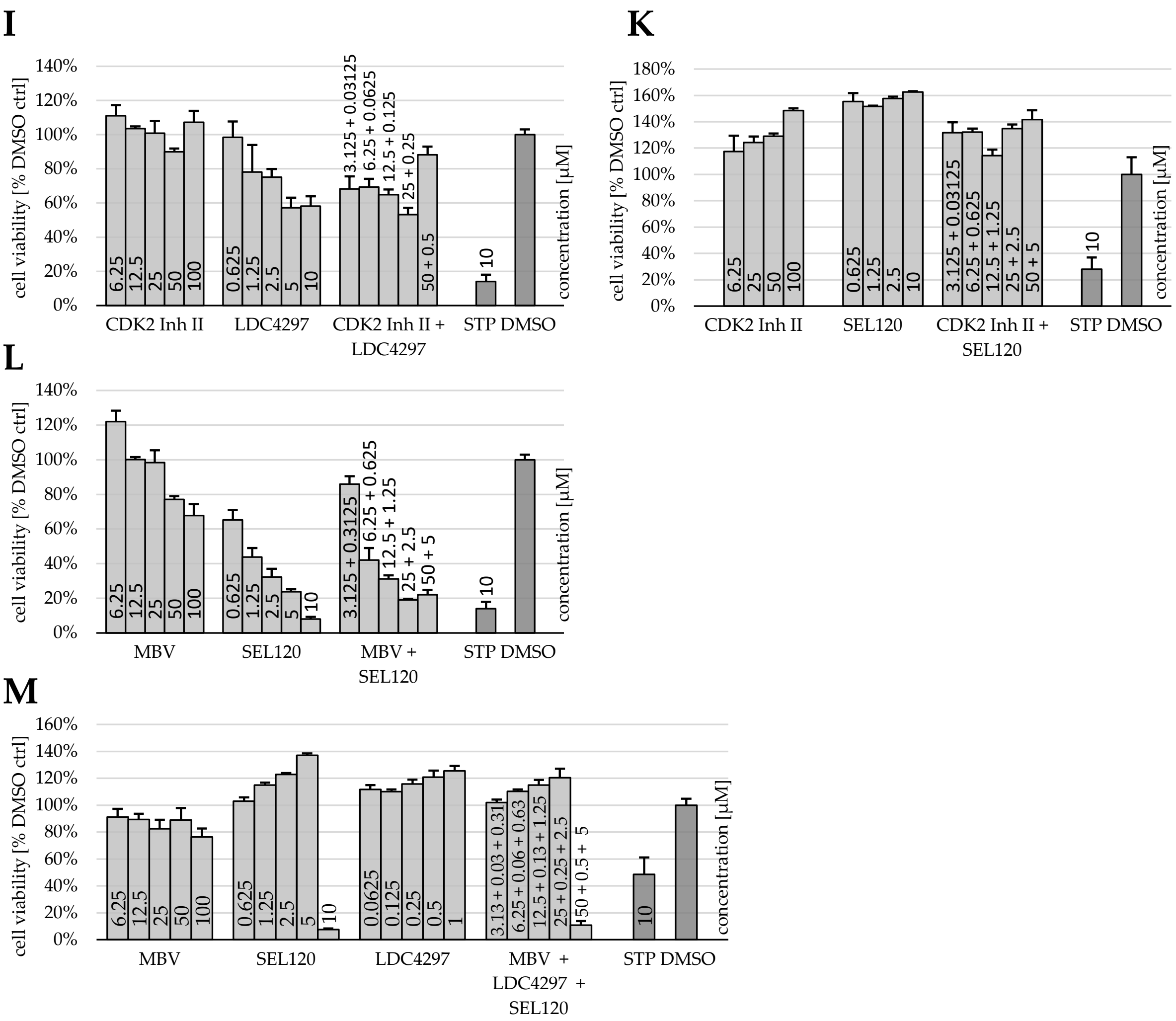


**Figure S1.** Assessment of cytotoxicity of various PKIs in single treatments and in combinations. **(A)** MBV + LDC4297, **(B)** Vi7392 + LDC4297, **(C)** Gö6976 + LDC4297, **(D)** Ax7396 + LDC4297, **(E)** SEL120 + LDC4297, **(F)** MBV + THAL-SNS032, **(G)** SEL120 + THAL-SNS032, **(H)** MBV + CDK2 Inh II, **(I)** CDK2 Inh II + LDC4297, **(K)** CDK2 Inh II + SEL120, **(L)** MBV + SEL120, **(M)** MBV + LDC4297 + SEL120. Staurosporine (STP) was used as a positive control (10 µM, added 3 h before harvesting); experiments were performed in biological triplicates and data are given as mean values + SD; ctrl, control.

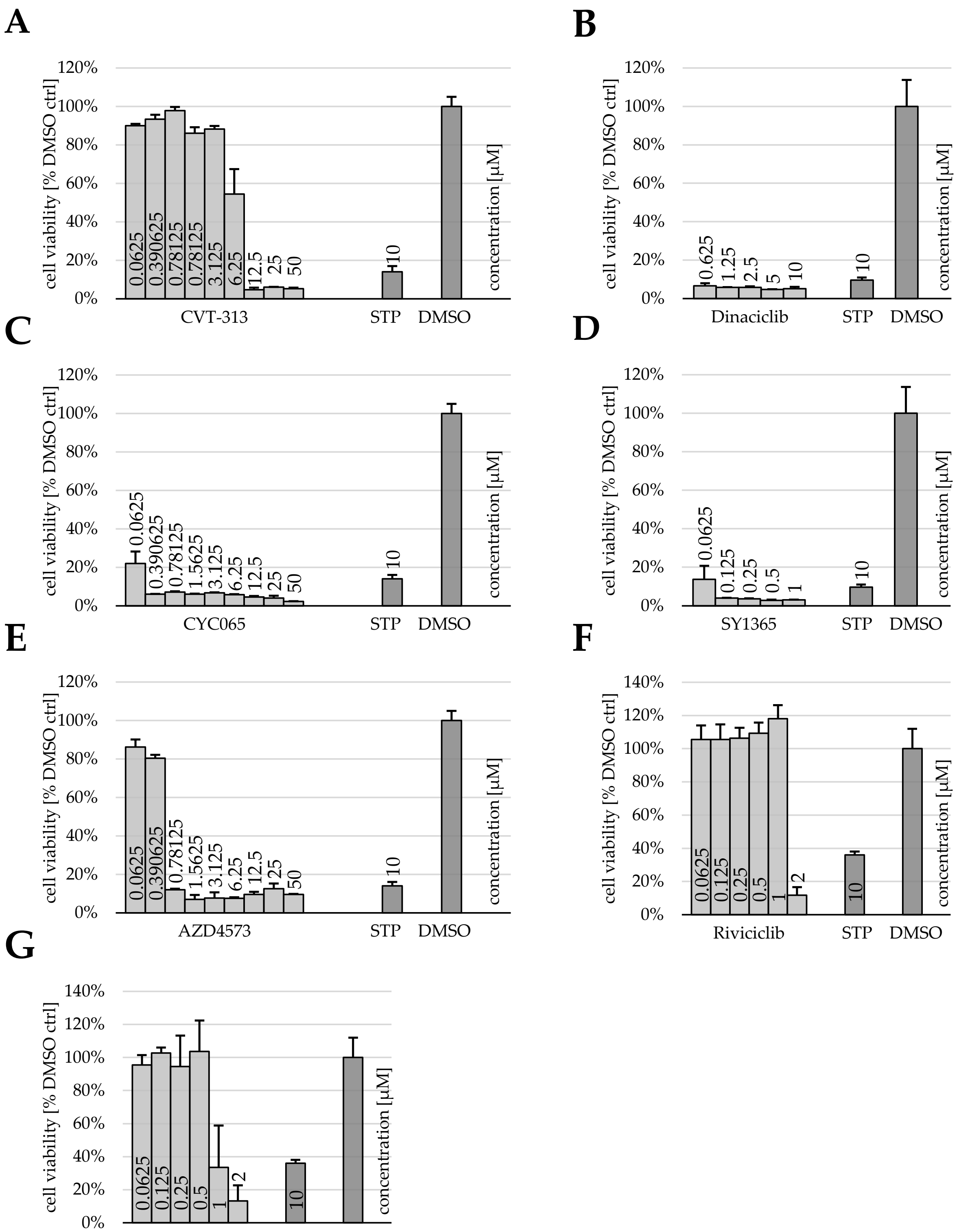


**Figure S2.** Assessment of cytotoxicity of distinct PKIs presently investigated in clinical trials. **(A)** CVT-313, **(B)** dinaciclib, **(C)** CYC065, **(D)** SY1365, **(E)** AZD4573, **(F)** riviciclib, **(G)** samuraciclib. Staurosporine (STP) was used as a positive control (10 µM, added 3 h before harvesting); experiments were performed in biological triplicates and data are given as mean values + SD; ctrl, control.

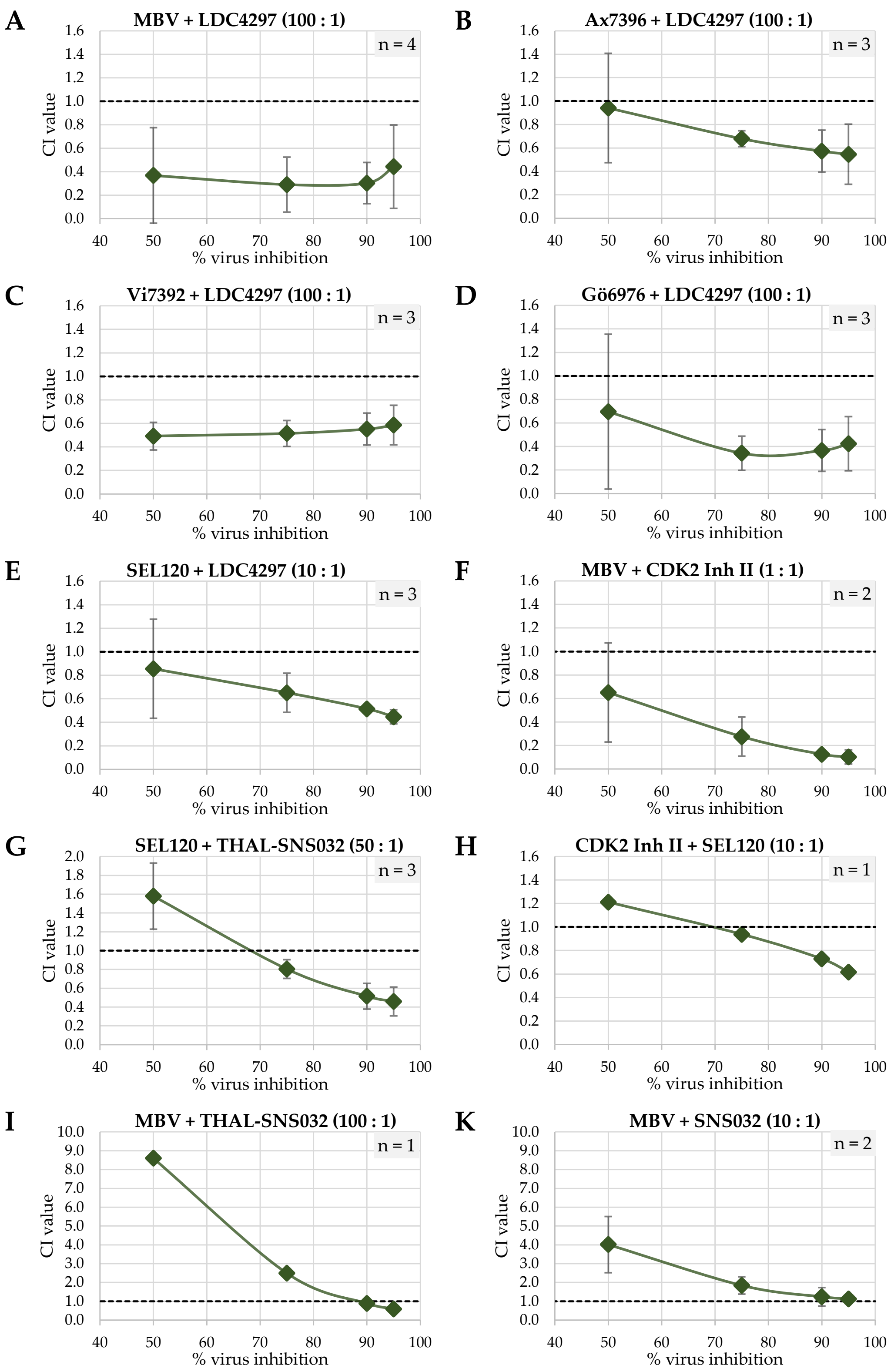


**Figure S3.** Assessment of PKI drug interactions utilizing the Loewe additivity fixed-dose assay. **(A)** MBV + LDC4297 (ratio 100 : 1), **(B)** Ax7396 + LDC4297 (100 : 1), **(C)** Vi7392 + LDC4297 (100 : 1), **(D)** Gö6976 + LDC4297 (100 : 1), **(E)** SEL120 + LDC4297 (10 : 1), **(F)** MBV + CDK2 Inh II (1: 1), **(G)** SEL120 + THAL-SNS032 (100 : 1), **(H)** CDK2 Inh II + SEl120 (10 : 1), **(I)** MBV + THAL-SNS032 (100 : 1), **(K)** MBV + SNS032 (10 : 1). Data are presented as mean CI values ± SD, as extrapolated at 50%, 75%, 90% and 95% virus inhibition across the number of individual experiments (n=1 to n=4). Individual experiments were performed as biological duplicates; CI, combination index.

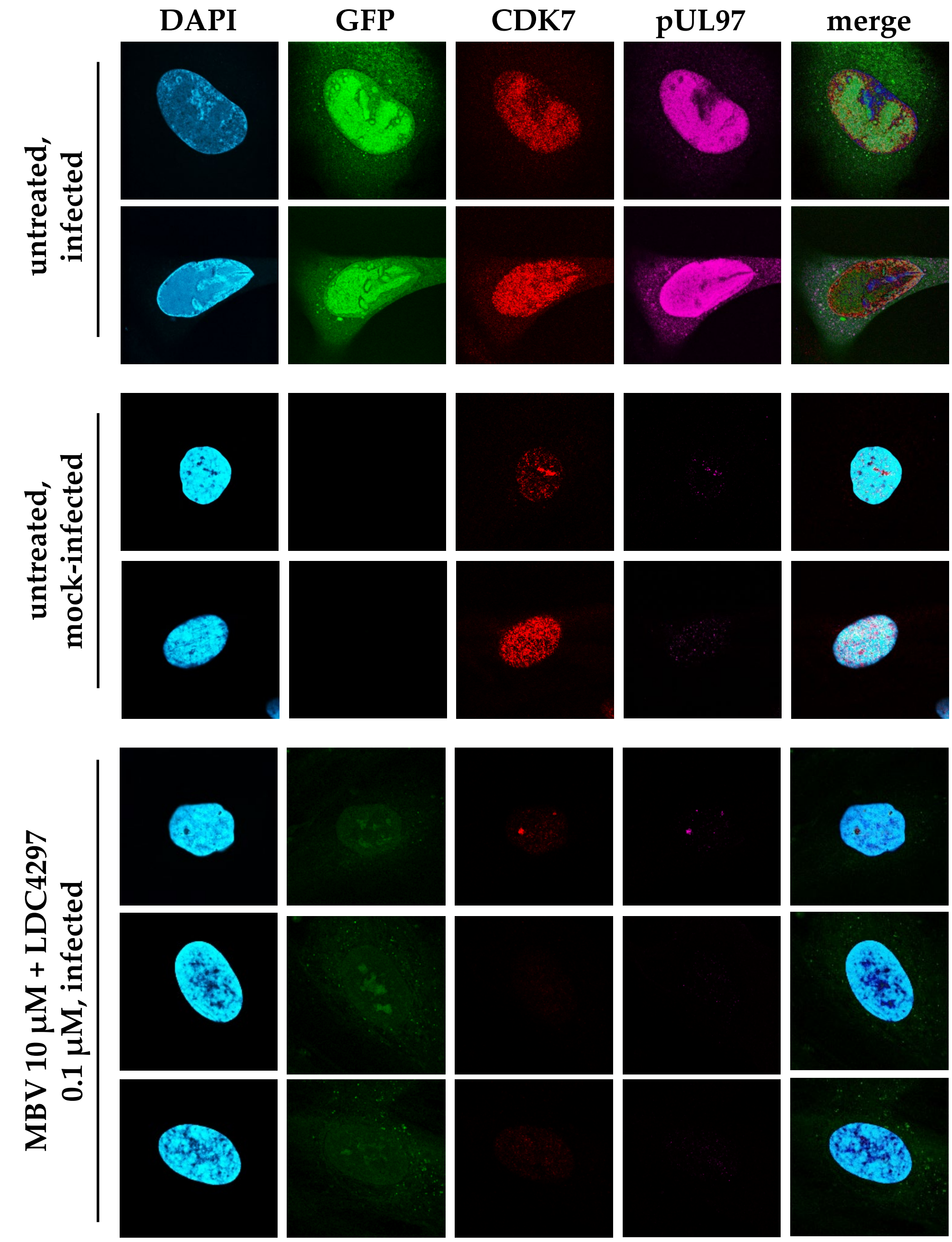


**Figure S4.** Assessment of the intracellular localization of CDK7 and pUL97 using confocal laser-scanning microscopic analysis. HFFs were seeded on glass slides, used for HCMV infection (AD169-GFP, MOI of 0.25 GFP-FU/ml) one day later or remained mock-infected and treated with the indicated concentrations of compounds for 7 days before fixation and staining. Cells were analyzed under the following conditions: drug-untreated, HCMV-infected (upper section); untreated, mock-infected (middle section); HCMV-infected, treated with a combination of MBV and LDC4297 (concentrations of 10 µM and 0.1 µM; lower section). Each section presents 2-3 exemplary cell samples to illustrate typical patterns of intracellular localization and the degree of visual variation.

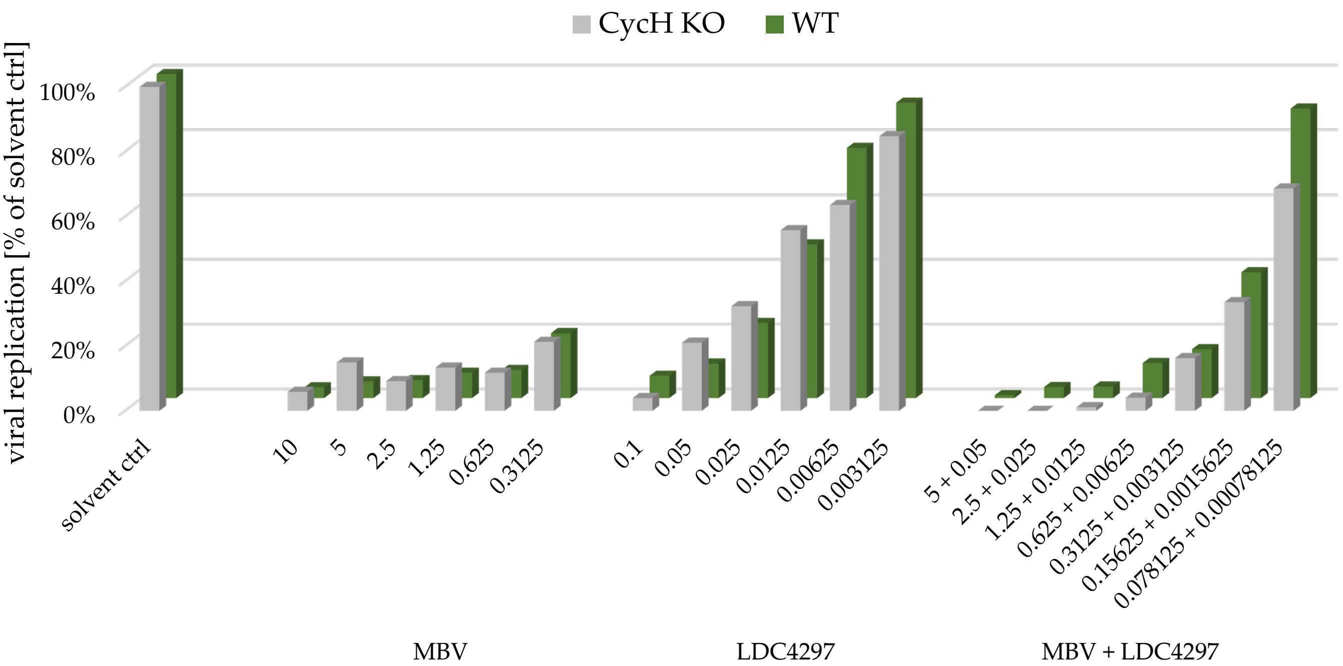


**Figure S5.** Assessment of antiviral efficacy of MBV, LDC4297 and the combination treatment MBV + LDC4297 in wild-type HFFs (WT) and partial cyclin H knock-out HFFs (CycH KO). HFFs were seeded in 12-well culture plates and treated with lentiviral supernatants to achieve a CRISPR/Cas9-mediated transient cyclin H knock-down. Subsequently, cells were directly infected with HCMV AD169-GFP for 90 min, then fresh medium, supplemented with indicated concentrations of compounds, was added. Cells were harvested and lysed 7 d post-infection and viral GFP reporter signals were measured in quadruplicate. Data are presented as mean values of biological duplicates, adjusted in percent to the respective solvent DMSO control (ctrl).

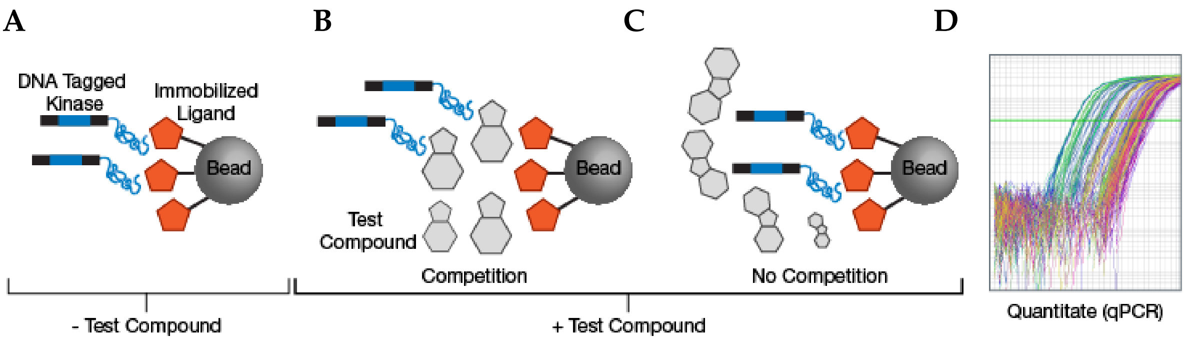


**Figure S6.** Assay principle of the DiscoverX KINOMEscan™. The immobilized ligand binds to the target kinase, which has been tagged with DNA (**A**). If the test compound (Vi7392) binds the kinase active site either directly (sterically) or indirectly (allosterically), kinase binding to the immobilized ligand will be reduced, lowering the amount of kinase captured on the bead (**B**). Conversely, if the tested kinase is not bound by Vi7392, no effect on the amount of kinase captured on the bead is detected (**C**). Screening hits are identified by measuring the amount of kinase captured in test versus control samples by using a quantitative, precise and ultra-sensitive qPCR method that detects the associated DNA label (**D**). In a similar manner, dissociation constants (Kds) for test compound-kinase interactions are calculated by measuring the amount of kinase captured on the solid support as a function of the test compound concentration. For methodological details, see *https://www.discoverx.com/services/drug-discovery-development-services/kinase-profiling/kinomescan*.

# Supplementary Tables

**Table S1.** Assessment of secondary cellular targets of pUL97 inhibitor Vi7392 by commercial DiscoverX KINOMEscan™ Profiling. Vi7392 was applied at a concentration of 3 µM. Data indicate two levels of cellular targets of Vi7392 on the basis of a drug competition-reduced binding, at a stringency of ≤ 10% (S10; see dark shadings) or lower stringency of ≤ 35% (S35; see light shadings). The following candidate kinases binding to Vi7392 were considered relevant: CDKL1 and PIP5K2C (S10); CDKL1, PIP5K2C, EGFR, MAPK2K5, MKNK2 and PRKG2 (S35; cancer-specific kinase mutants of EGFR and FLT3 without a known relevance for HCMV infection were not taken into consideration). For methodological details, see *https://www.discoverx.com/services/drug-discovery-development-services/kinase-profiling/kinomescan*.

| Vi7392 (3 µM) | | | | | |
|---|---|---|---|---|---|
| **Gene Symbol** | **%Ctrl** | **Gene Symbol** | **%Ctrl** | **Gene Symbol** | **%Ctrl** |
| AAK1 | 100 | BIKE | 100 | CDKL3 | 100 |
| ABL1(E255K)-phosphorylated | 80 | BLK | 91 | CDKL5 | 100 |
| ABL1(F317I)-nonphosphorylated | 85 | BMPR1A | 100 | CHEK1 | 100 |
| ABL1(F317I)-phosphorylated | 100 | BMPR1B | 100 | CHEK2 | 100 |
| ABL1(F317L)-nonphosphorylated | 100 | BMPR2 | 100 | CIT | 91 |
| ABL1(F317L)-phosphorylated | 100 | BMX | 96 | CLK1 | 75 |
| ABL1(H396P)-nonphosphorylated | 92 | BRAF | 100 | CLK2 | 100 |
| ABL1(H396P)-phosphorylated | 100 | BRAF(V600E) | 100 | CLK3 | 90 |
| ABL1(M351T)-phosphorylated | 100 | BRK | 100 | CLK4 | 90 |
| ABL1(Q252H)-nonphosphorylated | 76 | BRSK1 | 100 | CSF1R | 100 |
| ABL1(Q252H)-phosphorylated | 100 | BRSK2 | 100 | CSF1R-autoinhibited | 100 |
| ABL1(T315I)-nonphosphorylated | 100 | BTK | 100 | CSK | 100 |
| ABL1(T315I)-phosphorylated | 100 | BUB1 | 100 | CSNK1A1 | 64 |
| ABL1(Y253F)-phosphorylated | 100 | CAMK1 | 100 | CSNK1A1L | 100 |
| ABL1-nonphosphorylated | 50 | CAMK1B | 100 | CSNK1D | 100 |
| ABL1-phosphorylated | 51 | CAMK1D | 100 | CSNK1E | 63 |
| ABL2 | 66 | CAMK1G | 100 | CSNK1G1 | 100 |
| ACVR1 | 100 | CAMK2A | 96 | CSNK1G2 | 100 |
| ACVR1B | 100 | CAMK2B | 88 | CSNK1G3 | 84 |
| ACVR2A | 100 | CAMK2D | 100 | CSNK2A1 | 95 |
| ACVR2B | 100 | CAMK2G | 100 | CSNK2A2 | 100 |
| ACVRL1 | 100 | CAMK4 | 100 | CTK | 100 |
| ADCK3 | 100 | CAMKK1 | 100 | DAPK1 | 98 |
| ADCK4 | 100 | CAMKK2 | 100 | DAPK2 | 100 |
| AKT1 | 98 | CASK | 93 | DAPK3 | 90 |
| AKT2 | 100 | CDC2L1 | 98 | DCAMKL1 | 99 |
| AKT3 | 100 | CDC2L2 | 100 | DCAMKL2 | 100 |
| ALK | 100 | CDC2L5 | 97 | DCAMKL3 | 100 |
| ALK(C1156Y) | 100 | CDK11 | 89 | DDR1 | 59 |
| ALK(L1196M) | 100 | CDK2 | 100 | DDR2 | 100 |
| AMPK-alpha1 | 100 | CDK3 | 100 | DLK | 100 |
| AMPK-alpha2 | 97 | CDK4 | 100 | DMPK | 100 |
| ANKK1 | 100 | CDK4-cyclinD1 | 100 | DMPK2 | 100 |
| ARK5 | 100 | CDK4-cyclinD3 | 100 | DRAK1 | 86 |
| ASK1 | 86 | CDK5 | 100 | DRAK2 | 100 |
| ASK2 | 99 | CDK7 | 100 | DYRK1A | 100 |
| AURKA | 100 | CDK8 | 79 | DYRK1B | 78 |
| AURKB | 83 | CDK9 | 100 | DYRK2 | 100 |
| AURKC | 92 | CDKL1 | 6.2 | EGFR | 17 |
| AXL | 100 | CDKL2 | 100 | EGFR(E746-A750del) | 33 |

| colour legend | | |
|---|---|---|
| x < 10 | 10 ≤ x < 35 | x ≥ 35 |

Table S1. cont.

| Gene Symbol | %Ctrl | Gene Symbol | %Ctrl | Gene Symbol | %Ctrl |
|---|---|---|---|---|---|
| EGFR(G719C) | 7.8 | GAK | 76 | MAP3K15 | 90 |
| EGFR(G719S) | 9.6 | GCN2(Kin.Dom.2,S808G) | 100 | MAP3K2 | 100 |
| EGFR(L747-E749del, A750P) | 30 | GRK1 | 100 | MAP3K3 | 100 |
| EGFR(L747-S752del, P753S) | 38 | GRK2 | 100 | MAP3K4 | 92 |
| EGFR(L747-T751del,Sins) | 24 | GRK3 | 100 | MAP4K2 | 100 |
| EGFR(L858R) | 30 | GRK4 | 96 | MAP4K3 | 100 |
| EGFR(L858R,T790M) | 93 | GRK7 | 100 | MAP4K4 | 78 |
| EGFR(L861Q) | 24 | GSK3A | 100 | MAP4K5 | 100 |
| EGFR(S752-I759del) | 28 | GSK3B | 96 | MAPKAPK2 | 100 |
| EGFR(T790M) | 89 | HASPIN | 73 | MAPKAPK5 | 100 |
| EIF2AK1 | 78 | HCK | 89 | MARK1 | 100 |
| EPHA1 | 100 | HIPK1 | 54 | MARK2 | 100 |
| EPHA2 | 100 | HIPK2 | 100 | MARK3 | 99 |
| EPHA3 | 100 | HIPK3 | 97 | MARK4 | 100 |
| EPHA4 | 100 | HIPK4 | 81 | MAST1 | 100 |
| EPHA5 | 100 | HPK1 | 91 | MEK1 | 78 |
| EPHA6 | 100 | HUNK | 86 | MEK2 | 89 |
| EPHA7 | 100 | ICK | 100 | MEK3 | 80 |
| EPHA8 | 100 | IGF1R | 100 | MEK4 | 88 |
| EPHB1 | 100 | IKK-alpha | 100 | MEK5 | 15 |
| EPHB2 | 100 | IKK-beta | 100 | MEK6 | 75 |
| EPHB3 | 100 | IKK-epsilon | 70 | MELK | 96 |
| EPHB4 | 100 | INSR | 100 | MERTK | 100 |
| EPHB6 | 100 | INSRR | 100 | MET | 100 |
| ERBB2 | 44 | IRAK1 | 100 | MET(M1250T) | 97 |
| ERBB3 | 100 | IRAK3 | 100 | MET(Y1235D) | 71 |
| ERBB4 | 99 | IRAK4 | 100 | MINK | 58 |
| ERK1 | 100 | ITK | 67 | MKK7 | 100 |
| ERK2 | 100 | JAK1(JH1domain-catalytic) | 100 | MKNK1 | 95 |
| ERK3 | 100 | JAK1(JH2domain-pseudokinase) | 79 | MKNK2 | 25 |
| ERK4 | 94 | JAK2(JH1domain-catalytic) | 100 | MLCK | 74 |
| ERK5 | 100 | JAK3(JH1domain-catalytic) | 100 | MLK1 | 84 |
| ERK8 | 82 | JNK1 | 97 | MLK2 | 100 |
| ERN1 | 100 | JNK2 | 100 | MLK3 | 100 |
| FAK | 86 | JNK3 | 100 | MRCKA | 98 |
| FER | 100 | KIT | 100 | MRCKB | 100 |
| FES | 100 | KIT(A829P) | 100 | MST1 | 100 |
| FGFR1 | 100 | KIT(D816H) | 86 | MST1R | 100 |
| FGFR2 | 97 | KIT(D816V) | 100 | MST2 | 100 |
| FGFR3 | 100 | KIT(L576P) | 100 | MST3 | 100 |
| FGFR3(G697C) | 99 | KIT(V559D) | 93 | MST4 | 100 |
| FGFR4 | 100 | KIT(V559D,T670I) | 100 | MTOR | 98 |
| FGR | 100 | KIT(V559D,V654A) | 100 | MUSK | 100 |
| FLT1 | 99 | KIT-autoinhibited | 81 | MYLK | 98 |
| FLT3 | 100 | LATS1 | 100 | MYLK2 | 100 |
| FLT3(D835H) | 86 | LATS2 | 79 | MYLK4 | 91 |
| FLT3(D835V) | 18 | LCK | 100 | MYO3A | 100 |
| FLT3(D835Y) | 100 | LIMK1 | 100 | MYO3B | 100 |
| FLT3(ITD) | 97 | LIMK2 | 100 | NDR1 | 87 |
| FLT3(ITD,D835V) | 100 | LKB1 | 92 | NDR2 | 100 |
| FLT3(ITD,F691L) | 100 | LOK | 95 | NEK1 | 92 |
| FLT3(K663Q) | 100 | LRRK2 | 100 | NEK10 | 100 |
| FLT3(N841I) | 100 | LRRK2(G2019S) | 100 | NEK11 | 100 |
| FLT3(R834Q) | 100 | LTK | 100 | NEK2 | 100 |
| FLT3-autoinhibited | 100 | LYN | 100 | NEK3 | 100 |
| FLT4 | 100 | LZK | 100 | NEK4 | 100 |
| FRK | 82 | MAK | 100 | NEK5 | 100 |
| FYN | 91 | MAP3K1 | 100 | NEK6 | 100 |

| colour legend | | |
|---|---|---|
| x < 10 | 10 ≤ x < 35 | x ≥ 35 |

**Table S1. cont.**

| Gene Symbol | %Ctrl | Gene Symbol | %Ctrl | Gene Symbol | %Ctrl |
|---|---|---|---|---|---|
| NEK7 | 100 | PLK1 | 100 | SRMS | 100 |
| NEK9 | 100 | PLK2 | 100 | SRPK1 | 100 |
| NIK | 100 | PLK3 | 72 | SRPK2 | 100 |
| NIM1 | 100 | PLK4 | 100 | SRPK3 | 90 |
| NLK | 100 | PRKCD | 100 | STK16 | 100 |
| OSR1 | 100 | PRKCE | 100 | STK33 | 100 |
| p38-alpha | 100 | PRKCH | 100 | STK35 | 100 |
| p38-beta | 100 | PRKCI | 84 | STK36 | 100 |
| p38-delta | 100 | PRKCQ | 100 | STK39 | 100 |
| p38-gamma | 84 | PRKD1 | 100 | SYK | 100 |
| PAK1 | 100 | PRKD2 | 100 | TAK1 | 100 |
| PAK2 | 100 | PRKD3 | 100 | TAOK1 | 100 |
| PAK3 | 100 | PRKG1 | 99 | TAOK2 | 100 |
| PAK4 | 100 | PRKG2 | 14 | TAOK3 | 100 |
| PAK6 | 100 | PRKR | 72 | TBK1 | 55 |
| PAK7 | 100 | PRKX | 100 | TEC | 100 |
| PCTK1 | 100 | PRP4 | 92 | TESK1 | 100 |
| PCTK2 | 100 | PYK2 | 94 | TGFBR1 | 100 |
| PCTK3 | 100 | QSK | 100 | TGFBR2 | 100 |
| PDGFRA | 92 | RAF1 | 96 | TIE1 | 100 |
| PDGFRB | 100 | RET | 100 | TIE2 | 100 |
| PDPK1 | 100 | RET(M918T) | 100 | TLK1 | 100 |
| PFCDPK1(P.falciparum) | 87 | RET(V804L) | 100 | TLK2 | 75 |
| PFPK5(P.falciparum) | 100 | RET(V804M) | 100 | TNIK | 100 |
| PFTAIRE2 | 99 | RIOK1 | 100 | TNK1 | 100 |
| PFTK1 | 100 | RIOK2 | 97 | TNK2 | 100 |
| PHKG1 | 98 | RIOK3 | 100 | TNNI3K | 100 |
| PHKG2 | 86 | RIPK1 | 100 | TRKA | 100 |
| PIK3C2B | 100 | RIPK2 | 63 | TRKB | 100 |
| PIK3C2G | 100 | RIPK4 | 100 | TRKC | 100 |
| PIK3CA | 100 | RIPK5 | 100 | TRPM6 | 98 |
| PIK3CA(C420R) | 89 | ROCK1 | 100 | TSSK1B | 100 |
| PIK3CA(E542K) | 85 | ROCK2 | 100 | TSSK3 | 58 |
| PIK3CA(E545A) | 62 | ROS1 | 100 | TTK | 100 |
| PIK3CA(E545K) | 65 | RPS6KA4(Kin.Dom.1-N-terminal) | 94 | TXK | 96 |
| PIK3CA(H1047L) | 91 | RPS6KA4(Kin.Dom.2-C-terminal) | 100 | TYK2(JH1domain-catalytic) | 100 |
| PIK3CA(H1047Y) | 66 | RPS6KA5(Kin.Dom.1-N-terminal) | 100 | TYK2(JH2domain-pseudokinase) | 100 |
| PIK3CA(I800L) | 87 | RPS6KA5(Kin.Dom.2-C-terminal) | 100 | TYRO3 | 100 |
| PIK3CA(M1043I) | 100 | RSK1(Kin.Dom.1-N-terminal) | 100 | ULK1 | 100 |
| PIK3CA(Q546K) | 97 | RSK1(Kin.Dom.2-C-terminal) | 98 | ULK2 | 100 |
| PIK3CB | 100 | RSK2(Kin.Dom.1-N-terminal) | 100 | ULK3 | 92 |
| PIK3CD | 72 | RSK2(Kin.Dom.2-C-terminal) | 100 | VEGFR2 | 100 |
| PIK3CG | 100 | RSK3(Kin.Dom.1-N-terminal) | 100 | VPS34 | 100 |
| PIK4CB | 86 | RSK3(Kin.Dom.2-C-terminal) | 98 | VRK2 | 100 |
| PIKFYVE | 74 | RSK4(Kin.Dom.1-N-terminal) | 100 | WEE1 | 100 |
| PIM1 | 98 | RSK4(Kin.Dom.2-C-terminal) | 100 | WEE2 | 100 |
| PIM2 | 97 | S6K1 | 100 | WNK1 | 100 |
| PIM3 | 100 | SBK1 | 100 | WNK2 | 100 |
| PIP5K1A | 98 | SGK | 100 | WNK3 | 100 |
| PIP5K1C | 68 | SgK110 | 100 | WNK4 | 100 |
| PIP5K2B | 100 | SGK2 | 100 | YANK1 | 100 |
| PIP5K2C | 4.6 | SGK3 | 100 | YANK2 | 88 |
| PKAC-alpha | 100 | SIK | 100 | YANK3 | 100 |
| PKAC-beta | 97 | SIK2 | 100 | YES | 100 |
| PKMYT1 | 100 | SLK | 100 | YSK1 | 76 |
| PKN1 | 83 | SNARK | 100 | YSK4 | 100 |
| PKN2 | 100 | SNRK | 81 | ZAK | 100 |
| PKNB(M.tuberculosis) | 100 | SRC | 100 | ZAP70 | 100 |

| colour legend | | |
|---|---|---|
| x < 10 | 10 ≤ x < 35 | x ≥ 35 |
